# Population vs Individual Prediction of Poor Health From Results of Adverse Childhood Experiences Screening

**DOI:** 10.1001/jamapediatrics.2020.5602

**Published:** 2021-01-25

**Authors:** Jessie R. Baldwin, Avshalom Caspi, Alan J. Meehan, Antony Ambler, Louise Arseneault, Helen L. Fisher, HonaLee Harrington, Timothy Matthews, Candice L. Odgers, Richie Poulton, Sandhya Ramrakha, Terrie E. Moffitt, Andrea Danese

**Affiliations:** 1 Division of Psychology and Language Sciences, Department of Clinical, Educational and Health Psychology, University College London, London, United Kingdom; 2 Social, Genetic and Developmental Psychiatry Centre, Institute of Psychiatry, Psychology and Neuroscience, King’s College London, London, United Kingdom; 3 Department of Psychology and Neuroscience, Duke University, Durham, North Carolina; 4 PROMENTA, University of Oslo, Oslo, Norway; 5 Department of Psychiatry and Behavioral Sciences, Duke University, Durham, North Carolina; 6 Institute of Psychiatry, King's College London, London, United Kingdom; 7 Economic and Social Research Council Centre for Society and Mental Health, King’s College London, London, United Kingdom; 8 Department of Psychological Science, University of California, Irvine, Irvine; 9 Dunedin Multidisciplinary Health and Development Research Unit, Department of Psychology, University of Otago, Dunedin, New Zealand; 10 Institute of Psychiatry, Psychology and Neuroscience, Department of Child and Adolescent Psychiatry, King’s College London, London, United Kingdom; 11 National and Specialist Child and Adolescent Mental Health Services Trauma, Anxiety, and Depression Clinic, South London and Maudsley National Health Service Foundation Trust, London, United Kingdom

## Abstract

**Importance:**

Adverse childhood experiences (ACEs) are well-established risk factors for health problems in a population. However, it is not known whether screening for ACEs can accurately identify individuals who develop later health problems.

**Objective:**

To test the predictive accuracy of ACE screening for later health problems.

**Design, Setting, and Participants:**

This study comprised 2 birth cohorts: the Environmental Risk (E-Risk) Longitudinal Twin Study observed 2232 participants born during the period from 1994 to 1995 until they were aged 18 years (2012-2014); the Dunedin Multidisciplinary Health and Development Study observed 1037 participants born during the period from 1972 to 1973 until they were aged 45 years (2017-2019). Statistical analysis was performed from May 28, 2018, to July 29, 2020.

**Exposures:**

ACEs were measured prospectively in childhood through repeated interviews and observations in both cohorts. ACEs were also measured retrospectively in the Dunedin cohort through interviews at 38 years.

**Main Outcomes and Measures:**

Health outcomes were assessed at 18 years in E-Risk and at 45 years in the Dunedin cohort. Mental health problems were assessed through clinical interviews using the Diagnostic Interview Schedule. Physical health problems were assessed through interviews, anthropometric measurements, and blood collection.

**Results:**

Of 2232 E-Risk participants, 2009 (1051 girls [52%]) were included in the analysis. Of 1037 Dunedin cohort participants, 918 (460 boys [50%]) were included in the analysis. In E-Risk, children with higher ACE scores had greater risk of later health problems (any mental health problem: relative risk, 1.14 [95% CI, 1.10-1.18] per each additional ACE; any physical health problem: relative risk, 1.09 [95% CI, 1.07-1.12] per each additional ACE). ACE scores were associated with health problems independent of other information typically available to clinicians (ie, sex, socioeconomic disadvantage, and history of health problems). However, ACE scores had poor accuracy in predicting an individual’s risk of later health problems (any mental health problem: area under the receiver operating characteristic curve, 0.58 [95% CI, 0.56-0.61]; any physical health problem: area under the receiver operating characteristic curve, 0.60 [95% CI, 0.58-0.63]; chance prediction: area under the receiver operating characteristic curve, 0.50). Findings were consistent in the Dunedin cohort using both prospective and retrospective ACE measures.

**Conclusions and Relevance:**

This study suggests that, although ACE scores can forecast mean group differences in health, they have poor accuracy in predicting an individual’s risk of later health problems. Therefore, targeting interventions based on ACE screening is likely to be ineffective in preventing poor health outcomes.

## Key Points

**Question:**

Can screening for adverse childhood experiences (ACEs) accurately predict individual risk for later health problems?

**Findings:**

In 2 population-based birth cohorts (with a total of 2927 individuals) growing up 20 years and 20 000 km apart, ACE scores were associated with mean group differences in health problems independent of other information available to clinicians. However, ACE scores had low accuracy in predicting health problems at the individual level.

**Meaning:**

ACE scores can forecast mean group differences in later health problems; however, ACE scores have poor accuracy in identifying individuals at high risk for future health problems.

## Teaser

This cohort study uses data from 2 birth cohorts to test the predictive accuracy of adverse childhood experience screening for later health problems.

## Introduction

Adverse childhood experiences (ACEs) show a dose-response association with mental and physical health problems.^1,2,3,4^ To prevent health problems, ACE screening has been proposed to identify at-risk individuals who may benefit from health interventions.^5,6,7^ ACE screening in children has already been implemented in primary care clinics in the US,^8,9^ while adults are being screened for ACEs through population-based telephone health surveys in the US^10^ and through health care assessments in the UK.^11,12,13^ Such screening commonly involves administering a questionnaire assessing exposure to 10 ACEs: physical, sexual, and emotional abuse; emotional and physical neglect; domestic violence; and parental substance abuse, mental illness, separation, and incarceration.^1^ Individuals with high ACE scores are then referred for health interventions (eg, medical and mental health services)^14^ or provided with information about support services.^11,15^ However, concerns have been raised about the utility of ACE screening in preventing poor health outcomes^15,16,17,18,19^ because of unanswered questions about forecasting, incremental prediction, discrimination, and measurement.

With regard to forecasting, it is unclear whether ACE scores are associated with future health problems because (with notable exceptions^20,21,22^) previous research has largely been cross-sectional, linking adults’ reports of ACEs to their concurrent health problems.^3^ Without clear temporal separation between ACEs and health outcomes, it is unclear whether previous associations might reflect recall bias owing to concurrent health problems.^23,24^ If ACE scores cannot forecast later health problems, then ACE screening is uninformative for targeting preventive interventions.

With regard to incremental prediction, it is unclear whether ACE scores are associated with future health problems beyond other information typically available to clinicians, such as preexisting health problems or demographics, such as sex and socioeconomic disadvantage. If ACE scores are not associated with health problems beyond clinically available information, then ACE screening will not provide added value.

With regard to discrimination, it is unclear whether ACE scores differentiate between individuals who do and do not develop later health problems. Although previous research has found mean differences in health outcomes across groups of individuals with different ACE scores, individuals with the same ACE score have heterogeneous outcomes.^1,25^ If ACE scores do not accurately discriminate between individuals who do and do not develop health problems, allocating interventions on the basis of ACE scores might result in overreferrals of exposed individuals who will not develop health problems (false positives) and underreferrals of unexposed individuals who will develop health problems (false negatives).

With regard to measurement, it is unclear whether the ability of ACE scores to predict health outcomes differs depending on whether ACEs are assessed prospectively in childhood or retrospectively in adulthood. Indeed, prospective and retrospective measures of ACEs identify largely different groups of individuals^26^ and tend to have different associations with health outcomes.^2,27^ If the predictive ability of ACE scores differs based on prospective vs retrospective measurement, screening will have different utility based on the assessment method.

This study directly addressed these questions to inform policymakers and practitioners about the value of screening for ACEs in improving health. Using data from 2 population-representative birth cohorts, we examined forecasting by testing whether individuals with higher ACE scores had a greater mean risk of later mental and physical health problems. To examine incremental prediction, we tested whether individuals with higher ACE scores had a greater risk of later health problems independent of other clinically available information. To examine discrimination, we tested the predictive accuracy of ACE scores in identifying individuals with or without later health problems. To examine measurement, we tested the above questions using ACE scores assessed both prospectively in childhood and retrospectively in adulthood.

## Methods

A brief description of the samples and measures is below, and a full description is in eMethods 1-12 in the Supplement. The rationale for inclusion is in eMethods 1 in the Supplement, and the prevalence of all variables is described in eTable 1 in the Supplement. This project was preregistered.^28^ Analyses were checked for reproducibility by an independent data analyst, who recreated the code by working from the manuscript and applied it to a fresh data set. The R^29^ code is available online.^30^ This study follows the Strengthening the Reporting of Observational Studies in Epidemiology (STROBE) reporting guideline (eMethods 13 in the Supplement). A separate ethics approval was not required for this study because ethical approval was already granted for the analysis of data obtained during each assessment phase of the E-Risk and Dunedin cohorts.

### The Environmental Risk Longitudinal Twin Study

#### Sample

**Figure 1.**
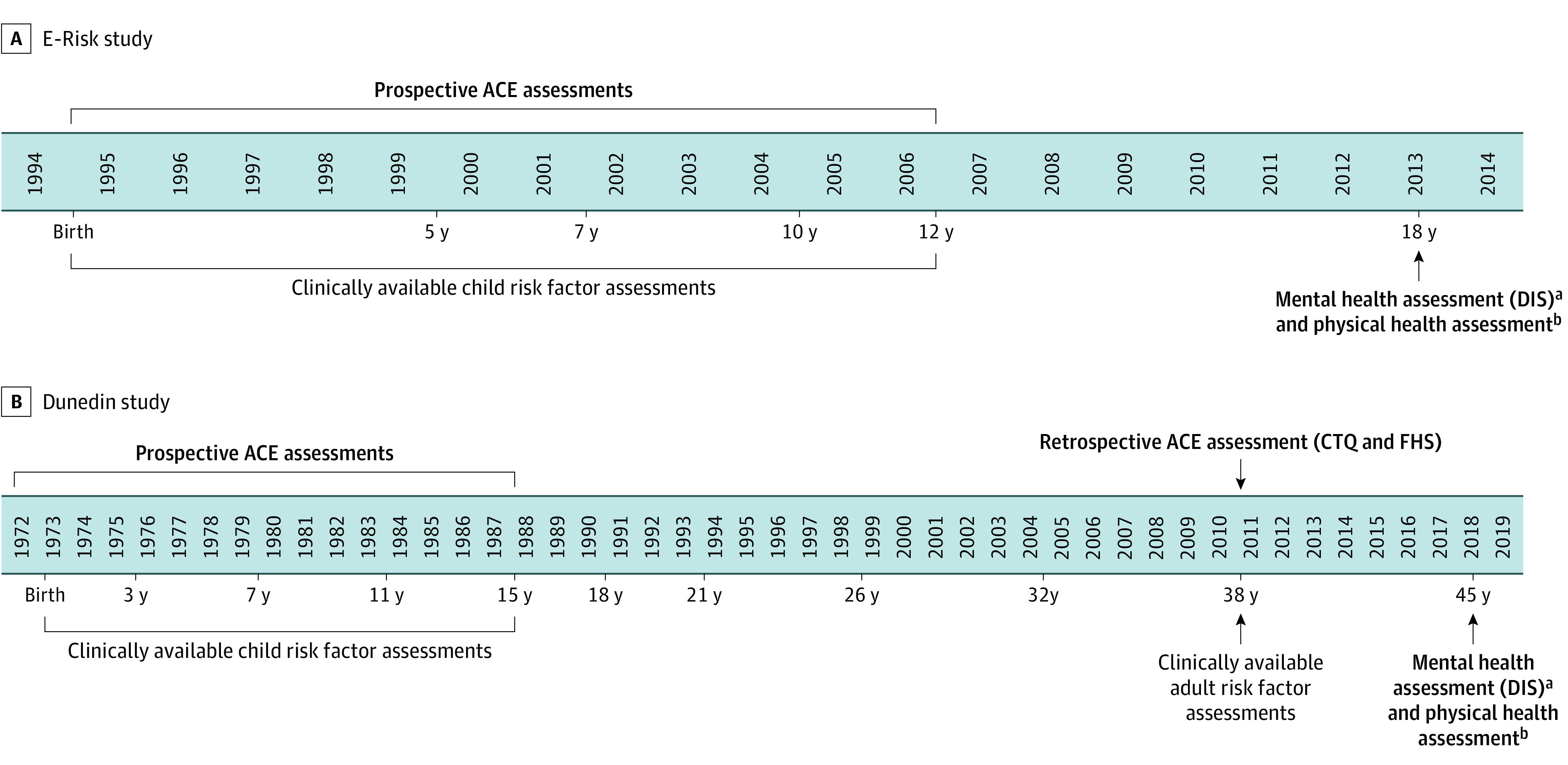
Timeline for Assessments of Adverse Childhood Experiences (ACEs) and Health in the Environmental Risk (E-Risk) Longitudinal Twin Study and the Dunedin Multidisciplinary Health and Development Study A, In the E-Risk study (N = 2232; 93% participation at 18 years), we examined whether prospectively assessed ACEs predicted mental and physical health problems at 18 years. Analyses testing incremental prediction controlled for clinically available childhood risk factors. B, In the Dunedin study (N = 1037; 94% participation at 45 years), we examined whether prospectively assessed ACEs predicted mental and physical health problems at 45 years. Analyses testing incremental prediction controlled for clinically available childhood risk factors. We also examined whether retrospectively assessed ACEs (measured at 38 years) predicted mental and physical health problems at 45 years. Analyses testing incremental prediction controlled for clinically available adult risk factors. CTQ indicates Childhood Trauma Questionnaire; DIS, Diagnostic Interview Schedule; and FHS, Family History Screen. ^a^Assessments of depression, anxiety, self-harm, suicide attempt, attention-deficit/hyperactivity disorder, alcohol dependence, and drug dependence in both cohorts were made through the DIS. ^b^In both cohorts, obesity was defined as a body mass index of 30 or higher (calculated as weight in kilograms divided by height in meters squared), inflammation was assessed via dried blood spots (in E-Risk study) or serum (in Dunedin study) and was defined as a C-reactive protein level higher than 0.3 mg/dL (to convert to milligrams per liter, multiply by 10), asthma was assessed through self-report, sexually transmitted diseases were assessed through self-report, sleep problems were defined as scores higher than 5 on the Pittsburgh Sleep Quality Index, and daily cigarette smoking was assessed through self-report.

The Environmental Risk (E-Risk) Longitudinal Twin Study tracks the development of a birth cohort of 2232 British children (Figure 1; eMethods 2 and eFigure 1 in the Supplement).^31^ The Joint South London and Maudsley and the Institute of Psychiatry Research Ethics Committee approved each phase of the study. Parents provided written informed consent, and twins provided written assent between 5 and 12 years of age and then provided informed consent at 18 years of age.

#### Measures

##### ACEs

Physical abuse, sexual abuse, emotional abuse and neglect, physical neglect, domestic violence, parental antisocial behavior, family history of substance abuse, family history of mental health problems, and parental separation or divorce between birth and age 12 years were assessed during 4 home visits when the children were aged 5 to 12 years (eMethods 3 and eFigure 2 in the Supplement).^32^ An ACE score was derived that summed the number of types of ACEs experienced.

##### Mental and Physical Health Problems at 18 Years

Depression, anxiety, self-harm, suicide attempt, attention-deficit/hyperactivity disorder (ADHD), alcohol dependence, and drug dependence at 18 years were assessed through private interviews with participants (eMethods 4 in the Supplement). Obesity, inflammation, asthma, sexually transmitted diseases (STDs), sleep problems, and smoking were assessed at 18 years (eMethods 5 in the Supplement).

##### Clinically Available Childhood Risk Factors

We collected information about children available to clinicians and known to be associated with later health. This information included sex, childhood family socioeconomic status, and childhood mental and physical health problems (eMethods 6 in the Supplement).

### The Dunedin Longitudinal Study

#### Sample

The Dunedin Longitudinal Study tracks a 1972-1973 birth cohort of 1037 children born in Dunedin, New Zealand (Figure 1; eMethods 7 and eFigure 3 in the Supplement).^33^ The University of Otago Ethics Committee approved each phase of the study. Participants provided written informed consent.

#### Measures

##### ACEs

Physical abuse, sexual abuse, emotional abuse, physical neglect, emotional neglect, domestic violence, incarceration of a family member, family history of substance abuse, family history of mental illness, loss of a parent, and parental separation or divorce were assessed prospectively and retrospectively (eMethods 8 and eFigure 2 in the Supplement).^2^ Prospective ACE scores were generated from records gathered during 7 biennial assessments carried out from 3 to 15 years, including social service contacts; structured notes from interviewers, pediatricians, psychometricians, and nurses who assessed study children and their parents; teachers’ notes of concern; and parental questionnaires. Retrospective ACE scores were ascertained through a structured interview at 38 years using the Childhood Trauma Questionnaire,^34^ the Family History Screen,^35^ and additional questions.

##### Mental and Physical Health Problems at 45 Years

To match outcomes in the E-Risk study, depression, anxiety, self-harm, suicide attempt, ADHD, alcohol dependence, and drug dependence were assessed at 45 years through private interviews with participants (eMethods 9 in the Supplement). Obesity, inflammation, asthma, STDs, sleep problems, and smoking were also assessed at 45 years (eMethods 10 in the Supplement).

##### Clinically Available Health Risk Factors

Childhood risk factors included sex, childhood family socioeconomic status, and childhood mental and physical health problems (eMethods 11 in the Supplement). Adult risk factors included sex, socioeconomic status, and self-reported health at 38 years (eMethods 12 in the Supplement).

### Statistical Analysis

#### E-Risk Study

Statistical analysis was performed from May 28, 2018, to July 29, 2020. To test whether prospectively ascertained ACEs experienced between birth and 12 years forecasted health problems at 18 years, we used quasi-Poisson generalized linear models^36^ to obtain relative risks for any mental health problem, any physical health problem, and individual mental and physical health problems. We obtained robust SEs to account for familial clustering. To test whether prospectively ascertained ACE scores incrementally predicted health problems at 18 years above other clinically available childhood risk factors (sex, socioeconomic disadvantage, and childhood mental or physical health problems), we expanded the quasi-Poisson generalized linear models to include these covariates.

To test whether prospectively ascertained ACE scores discriminated between young adults with and without health problems, we used receiver operating characteristic curve analyses, which yield an area under the curve (AUC) statistic, indexing the probability that a random participant with a health problem at 18 years had a higher ACE score than a participant without a health problem. Values can range between 0.50 (chance) and 1.00 (perfect discrimination), with suggested grading as fail or very poor (0.5-0.6), poor (0.6-0.7), fair (0.7-0.8), good (0.8-0.9), and excellent (0.9-1.0).^37^ We conducted 2 sensitivity analyses: (1) using a binary ACE measure comparing 4 or more ACEs vs 3 or fewer ACEs to test this commonly used cutoff^14^; and (2) rerunning analyses in 10 subsamples comprising one randomly selected twin per pair to test the role of familial clustering.

#### Dunedin Study

Statistical analysis was performed from May 28, 2018, to July 29, 2020. To test whether the findings were replicated in an independent cohort of older individuals, we repeated the above analyses in the Dunedin Study. Here we tested whether prospectively ascertained ACEs experienced between birth and 15 years were associated with health problems at 45 years. To test whether the findings differed when using retrospective rather than prospective measurement of ACEs, we tested whether ACE scores obtained from retrospective self-reports at 38 years were associated with health problems at 45 years.

## Results

### The E-Risk Study

#### Forecasting

**Figure 2.**
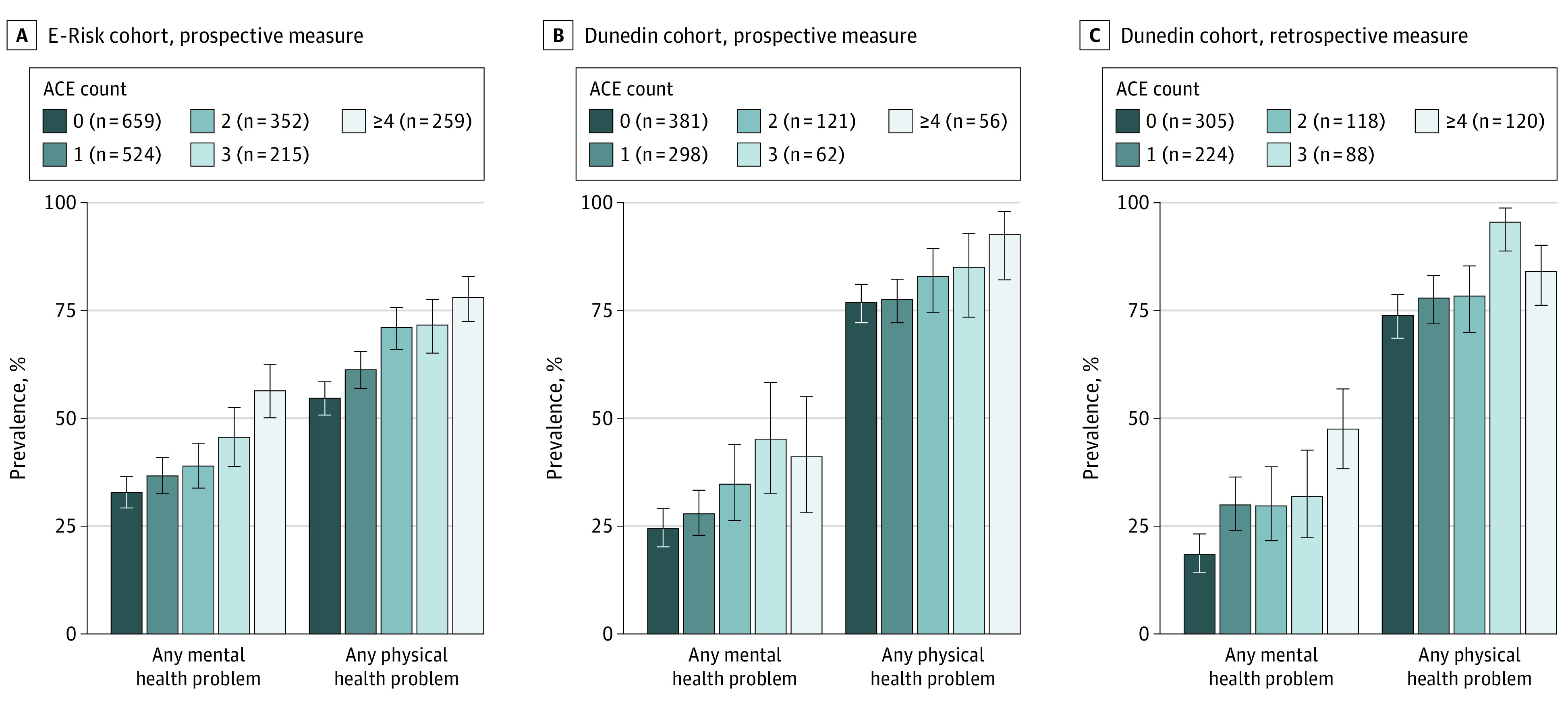
Prevalence of Health Problems in the Environmental Risk Longitudinal Twin (E-Risk) Study and Dunedin Multidisciplinary Health and Development Study Cohorts According to Adverse Childhood Experience (ACE) Score A, The prevalence of health problems at 18 years in the E-Risk cohort, as assessed with a prospective ACE measure. B, The prevalence of health problems at 45 years in the Dunedin cohort, as assessed with a prospective ACE measure. C, The prevalence of health problems at 45 years in the Dunedin cohort, as assessed with a retrospective ACE measure. The sample size as reported in the legend varies according to the health outcome (as reported fully in eTable 1 in the Supplement). Error bars indicate 95% CIs.

Of 2232 E-Risk participants, 2009 (1051 girls [52%]) were included in the analysis. Children who experienced more ACEs had greater risk of a mental health problem at 18 years (relative risk, 1.14 [95% CI, 1.10-1.18] per each additional ACE; Table, model 1).^36^ For example, 146 of 259 children (56%) exposed to 4 or more ACEs had a mental health problem vs 216 of 659 nonexposed children (33%) (Figure 2A). Sensitivity analyses showed that ACE scores were also associated with each individual mental health problem (eFigure 4A and eTable 2A, model 1, in the Supplement). Children who experienced more ACEs also had elevated risk of a physical health problem at 18 years (relative risk; 1.09 [95% CI, 1.07-1.12] per each additional ACE; Table, model 1),^36^ with 202 of 259 children (78%) exposed to 4 or more ACEs having a physical health problem vs 360 of 659 nonexposed children (55%) (Figure 2A). This risk generalized across all individual physical health problems (eFigure 5A and eTable 3A, model 1, in the Supplement).

**Table.** Association Between ACEs and Health Problems in the E-Risk and Dunedin Cohorts^a^

Cohort: ACE measure	No.	Relative risk (95% CI)				
		Model 1 (unadjusted)	Model 2 (adjusted for sex)	Model 3 (adjusted for SES at ACE assessment)	Model 4 (adjusted for health at ACE assessment)	Model 5 (adjusted for all risk factors)
E-Risk cohort (18 y)—prospective ACE measure						
Any mental health problem	2009	1.14 (1.10-1.18)	1.14 (1.10-1.18)	1.12 (1.08-1.17)	1.11 (1.07-1.15)	1.10 (1.06-1.15)
Any physical health problem	2009	1.09 (1.07-1.12)	1.10 (1.07-1.12)	1.07 (1.04-1.09)	1.08 (1.06-1.11)	1.06 (1.04-1.09)
Dunedin cohort (45 y)—prospective ACE measure						
Any mental health problem	918	1.17 (1.08-1.27)	1.17 (1.08-1.27)	1.17 (1.07-1.27)	1.15 (1.06-1.25)	1.15 (1.06-1.25)
Any physical health problem	872	1.04 (1.01-1.07)	1.04 (1.01-1.07)	1.03 (1.00-1.06)	1.04 (1.01-1.07)	1.03 (1.00-1.06)
Dunedin cohort (45 y)—retrospective ACE measure						
Any mental health problem	855	1.23 (1.14-1.31)	1.23 (1.14-1.32)	1.20 (1.12-1.29)	1.21 (1.12-1.30)	1.19 (1.10-1.28)
Any physical health problem	859	1.05 (1.02-1.07)	1.05 (1.02-1.07)	1.04 (1.01-1.06)	1.03 (1.01-1.06)	1.02 (1.00-1.05)

Abbreviations: ACE, adverse childhood experience; Dunedin, Dunedin Multidisciplinary Health and Development Study; E-Risk, Environmental Risk Longitudinal Twin Study; SES, socioeconomic status.

^a^ Results are presented as relative risks and 95% CIs for health problems per additional ACE experienced. We controlled for covariates measured at the time of ACE assessment to reflect information clinicians would have access to at the time of ACE screening; analyses using prospective ACE measures adjusted for risk factors measured in childhood (eg, family SES disadvantage and child mental health problems), whereas analyses using the retrospective ACE measure adjusted for risk factors in adulthood (eg, SES disadvantage at 38 years and self-reported health problems at 38 years). We adjusted for sex in analyses based on both prospective and retrospective ACE measures. The sample size for each outcome includes individuals with complete data for ACEs, the health outcome, and all covariates (eg, sex, SES, and prior health measures). Estimates were obtained from quasi-Poisson regression models, which are recommended vs binomial regression models to avoid convergence problems.^36^ However, findings were consistent with those obtained from logistic regression models (presented in eTable 6 in the Supplement).

#### Incremental Prediction

After accounting for risk factors typically available to clinicians (eg, sex, socioeconomic disadvantage, and prior health problems), children who experienced more ACEs still had greater risk of a mental health problem (relative risk, 1.10 [95% CI, 1.06-1.15]; Table, model 5),^36^ including all individual mental health problems (eTable 2A, model 5, in the Supplement). Children who experienced more ACEs also had greater risk of a physical health problem (relative risk, 1.06 [95% CI, 1.04-1.09]; Table, model 5),^36^ particularly sleep problems, STDs, and smoking (eTable 3A, model 5, in the Supplement).

#### Discrimination

**Figure 3.**
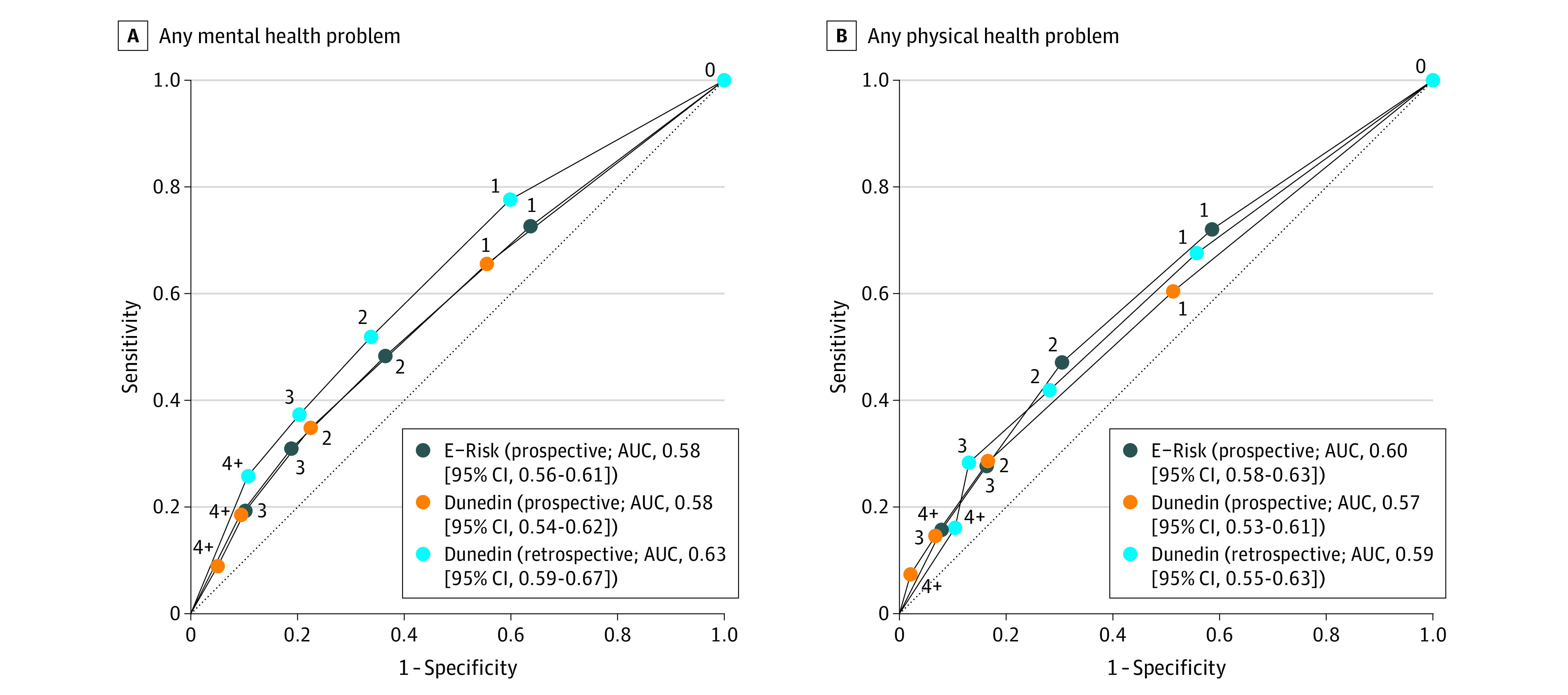
Predictive Accuracy for Health Problems Based on Adverse Childhood Experience (ACE) Scores in the Environmental Risk (E-Risk) Longitudinal Twin Study and Dunedin Multidisciplinary Health and Development Study Cohorts A, Any mental health problem. B, Any physical health problem. The numbers on the lines indicate the number of ACEs. The lines display receiver operating characteristic curves with cutoffs for each ACE score. The dotted diagonal line indicates discrimination at chance level. Corresponding positive and negative likelihood ratios for the prediction of individual health outcomes by ACE scores are presented in eTable 7 in the Supplement. AUC indicates area under the curve.

ACE scores had very poor accuracy in predicting which children had a mental health problem at 18 years, with an AUC of 0.58 (95% CI, 0.56-0.61; Figure 3A). This AUC represents a 58% probability (ie, 8% above chance) that a random participant who developed a mental health problem had a higher ACE score than a random participant who did not. Discrimination was most accurate for drug dependence (AUC, 0.66 [95% CI, 0.60-0.71]) and least accurate for anxiety (AUC, 0.56 [95% CI, 0.51-0.61]) (eFigure 6A in the Supplement). ACE scores also showed poor accuracy in predicting which children had a physical health problem at 18 years (AUC, 0.60 [95% CI, 0.58-0.63]; Figure 3B). Discrimination was most accurate for smoking (AUC, 0.65 [95% CI, 0.62-0.68]) and least accurate for asthma (AUC, 0.54 [95% CI, 0.50-0.57]) (eFigure 7A in the Supplement). Predictive accuracy was similar based on a cutoff of 4 or more ACEs (eTable 4A in the Supplement) and was not explained by familial clustering (eTable 5 in the Supplement).

### Replication in the Dunedin Study

#### Forecasting

We next tested whether these findings replicated in an independent and older cohort with both prospective and retrospective ACE measures. Of 1037 Dunedin cohort participants, 918 (460 boys [50%]) were included in the analysis. In the Dunedin Study, children who experienced more ACEs had greater risk of a mental health problem at 45 years (relative risk, 1.17 [95% CI, 1.08-1.27]; Table, model 1^36^; Figure 2B). The risk generalized across several individual mental health problems (eFigure 4B and eTable 2B, model 1, in the Supplement). Children who experienced more ACEs also had greater risk of a physical health problem at 45 years (relative risk, 1.04 [95% CI, 1.01-1.07]; Table, model 1^36^; Figure 2B), particularly obesity, inflammation, and smoking (eFigure 5B and eTable 3B, model 1, in the Supplement).

#### Incremental Prediction

After accounting for sex, family socioeconomic disadvantage, and history of health problems, children who experienced more ACEs still had higher risk of a later mental health problem (relative risk, 1.15 [95% CI, 1.06-1.25]; Table, model 5),^36^ including several individual mental health problems (eTable 2B, model 5, in the Supplement). Independent of these clinically available risk factors, children who experienced more ACEs also had greater risk of a physical health problem (relative risk, 1.03 [95% CI, 1.00-1.06]; Table, model 5),^36^ particularly obesity and smoking (eTable 3B, model 5, in the Supplement).

#### Discrimination

Prospectively ascertained ACE scores had very poor accuracy in predicting which children had a mental health problem at 45 years (AUC, 0.58 [95% CI, 0.54-0.62]; Figure 3A). Prediction was most accurate for ADHD (AUC, 0.62 [95% CI, 0.52-0.72]) and least accurate for self-harm (AUC, 0.54 [95% CI, 0.43-0.65]) (eFigure 6B in the Supplement). Prospectively ascertained ACE scores also showed very poor accuracy in predicting which children had a physical health problem at 45 years (AUC, 0.57 [95% CI, 0.53-0.61]; Figure 3B). Prediction was most accurate for smoking (AUC, 0.65 [95% CI, 0.61-0.69]) and least accurate for sleep problems (AUC, 0.52 [95% CI, 0.48-0.55]) (eFigure 7B in the Supplement). Findings were consistent based on a cutoff measure of 4 or more ACEs (eTable 4B in the Supplement).

### Sensitivity Analyses With Retrospective Reports of ACEs in the Dunedin Study

To test whether screening adults retrospectively for ACEs could forecast later health problems, we replaced the prospective ACE measure with participants’ retrospective reports of ACEs at 38 years. As previously reported,^2^ agreement between prospective and retrospective measures was only moderate (*r* = 0.47; κ = 0.31).

Regarding forecasting, adults who retrospectively reported more ACEs at 38 years had greater risk of having mental and physical health problems at 45 years (Figure 2C; Table, model 1^36^). The risk generalized across all mental health problems (eFigure 4C and eTable 2C, model 1, in the Supplement) and to obesity, sleep problems, and smoking (eFigure 5C and eTable 3C, model 1, in the Supplement). Regarding incremental prediction, adults who retrospectively reported more ACEs still had greater risk of a mental health problem and slightly higher risk of a physical health problem after accounting for risk factors measured at the time of ACE assessment (sex, socioeconomic disadvantage, and self-reported health at 38 years; Table, model 5).^36^

Regarding discrimination, retrospectively ascertained ACE scores had poor accuracy in predicting which adults had a later mental health problem (AUC, 0.63 [95% CI, 0.59-0.67]; Figure 3A) or a physical health problem (AUC, 0.59 [95% CI, 0.55-0.63]; Figure 3B) at 45 years. For mental health, discrimination was most accurate for suicide attempt (AUC, 0.74 [95% CI, 0.60-0.88]) and least accurate for alcohol dependence (AUC, 0.56 [95% CI, 0.50-0.62]) (eFigure 6C in the Supplement). For physical health, discrimination was most accurate for smoking (AUC, 0.65 [95% CI, 0.60-0.69]) and least accurate for STDs (AUC, 0.51 [95% CI, 0.43-0.59]) (eFigure 7C in the Supplement). Predictive accuracy was similar based on a cutoff measure of 4 or more ACEs (eTable 4C in the Supplement).

## Discussion

We examined the clinical utility of screening for ACEs for the prediction of poor health outcomes in 2 birth cohorts growing up 20 years and 20 000 km apart. Our findings support previous cross-sectional research showing an association between ACEs and health problems^1,2,3^ and add novel insights.

First, to understand whether ACE scores could forecast future health problems, we capitalized on longitudinal prospective data in which ACEs were assessed before health outcomes. We found that individuals with higher ACE scores had, on average, elevated risk of later health problems, with each additional ACE forecasting 14% or more greater risk for mental health problems and 4% or more greater risk for physical health problems. These findings were consistent when health outcomes were assessed in adolescence (in E-Risk) and middle age (in Dunedin) despite the prevalence of such outcomes differing between the 2 time periods.

Second, to understand whether ACE screening could provide added value in predicting poor health, we tested whether ACE scores were associated with health problems above and beyond information typically available to clinicians (ie, sex, socioeconomic status, and history of health problems). We found that individuals with higher ACE scores had, on average, elevated risk of later health problems independent of other key risk factors.

Third, to understand whether ACE screening could accurately identify individuals at risk of poor health, we tested how well ACE scores discriminated between participants with or without later health problems. We observed low predictive accuracy, as the probability that a random individual with any mental or physical health problem had a higher ACE score than a random individual without a health problem was just above chance (AUCs ranging from 0.57 to 0.63). Although retrospective reports of ACEs predicted suicide attempts with fair accuracy (AUC, 0.74), predictive accuracy was generally poor when specific health problems were examined individually.

Fourth, because ACE screening is being recommended in both children and adults,^10,11,14^ we tested the predictive ability of ACE scores measured both prospectively in childhood and retrospectively in adulthood. Findings were consistent regardless of the ACE measure used, which suggests that screening both children and adults for ACEs has limited ability to inform individual prediction of poor health outcomes.

### Limitations

This research has limitations. First, the measures used to prospectively assess ACEs (repeated interviews, observations, and medical records) do not mirror the ACE screening methods used in clinical settings (ie, a single questionnaire). Nevertheless, findings based on our prospective ACE measures were consistent with those based on a single retrospective ACE assessment. Second, this study cannot inform about the predictive ability of ACE assessments more comprehensive than the ones in current use (eg, indexing frequency, timing, and duration of exposure, or spanning the whole adolescent period). Third, the discrimination accuracy estimates obtained (AUCs) are likely to be overoptimistic because they are based on models that provide the best fit for these data.^38^ Fourth, these findings from 2 population-based cohorts from the UK and New Zealand may not generalize to other populations. However, we observed a similar prevalence of ACEs and strength of associations between ACEs and health outcomes as found elsewhere.^1,3,39^ Fifth, these findings do not inform about the effectiveness of screening for broader social determinants of health^40^ or other traumas.^41^

## Conclusions

Despite these limitations, our findings can inform policymakers and practitioners about the value of screening for ACEs in predicting health outcomes. On the one hand, high ACE scores can identify groups of individuals at heightened mean risk of poor health later in life, independent of other clinical risk factors and regardless of whether ACEs were measured prospectively in childhood or retrospectively in adulthood. Therefore, our findings provide further support that ACEs are robust risk factors for ill health and that prevention of ACEs^42^ might relieve a broad health burden in the population. ACE screening could help reduce the persistence of ACEs if effective interventions are available to protect children identified as exposed.

On the other hand, ACE scores alone do not accurately discriminate between individuals with or without health problems in later life. Many individuals with high ACE scores will not develop poor health outcomes, and most poor health outcomes in the population will be observed in those with low ACE scores, as these groups are more prevalent. Therefore, these findings caution against the deterministic use of ACE scores in disease prediction and clinical decision-making. However, more research is needed to establish whether ACE scores can be used alongside other clinically available information to accurately predict individual poor health outcomes.^43^

## Notes

Supplement. **eMethods 1.** Rationale for Including Selected Health Outcomes **eMethods 2.** The E-Risk Study: Study Sample **eMethods 3.** The E-Risk Study: Adverse Childhood Experiences (ACEs) **eMethods 4.** The E-Risk Study: Mental Health Problems at Age 18 **eMethods 5.** The E-Risk Study: Physical Health Problems at Age 18 **eMethods 6.** The E-Risk Study: Clinically Available Childhood Risk Factors **eMethods 7.** The Dunedin Study: Study Sample **eMethods 8.** The Dunedin Study: Adverse Childhood Experiences (ACEs) **eMethods 9.** The Dunedin Study: Mental Health Problems at Age 45 **eMethods 10.** The Dunedin Study: Physical Health Problems at Age 45 **eMethods 11.** The Dunedin Study: Clinically Available Childhood Risk Factors **eMethods 12.** The Dunedin Study: Clinically Available Adult Risk Factors **eMethods 13.** STROBE Statement—Checklist of Items That Should be Included in Reports of Cohort Studies **eTable 1.** Analytic Sample Sizes and Prevalence of Health Outcomes by ACE Score **eTable 2.** Association Between ACEs and Individual Mental Health Problems in the E-Risk and Dunedin Cohorts **eTable 3.** Association Between ACEs and Individual Physical Health Problems in the E-Risk and Dunedin Cohorts **eTable 4.** Prediction Accuracy for Health Problems Based on ACE Cut-Off Score of 4+ vs 0-3 ACEs **eTable 5.** Sensitivity Analysis for Prediction Accuracy in the E-Risk Cohort Testing Potential Bias Arising From Clustered (Twin) Data **eTable 6.** Association Between ACEs and Health Problems in the E-Risk and Dunedin Cohorts Based on Logistic Regression Models **eTable 7.** Likelihood Ratios Indexing the Predictive Ability of ACE Scores for Health Problems in the E-Risk and Dunedin Cohorts **eFigure 1.** Population Representativeness of the E-Risk Study **eFigure 2.** Prevalence of ACEs in the E-Risk and Dunedin Cohorts in Comparison to the CDC ACEs Study **eFigure 3.** Attrition Analysis in the Dunedin Study **eFigure 4.** Prevalence of Mental Health Problems in the E-Risk Cohort and in the Dunedin Cohort According to ACE Score **eFigure 5.** Prevalence of Physical Health Problems in the E-Risk Cohort and in the Dunedin Cohort According to ACE Score **eFigure 6.** Predictive Accuracy for Individual Mental Health Problems Based on ACE Scores in the E-Risk and Dunedin Cohorts **eFigure 7.** Predictive Accuracy for Individual Physical Health Problems Based on ACE Scores in the E-Risk and Dunedin Cohorts **eReferences**
