# Supplementary material for: Population vs Individual Prediction of Poor Health From Results of Adverse Childhood Experiences Screening
Source: JAMA Pediatr. 2021 Jan 25;175(4):1–9. doi: 10.1001/jamapediatrics.2020.5602 (PMC7835926; doi:10.1001/jamapediatrics.2020.5602)
Supplement: Supplement. — eMethods 1. Rationale for Including Selected Health Outcomes eMethods 2. The E-Risk Study: Study Sample eMethods 3. The E-Risk Study: Adverse Childhood Experiences (ACEs) eMethods 4. The E-Risk Study: Mental Health Problems at Age 18 eMethods 5. The E-Risk Study: Physical Health Problems at Age 18 eMethods 6. The E-Risk Study: Clinically Available Childhood Risk Factors eMethods 7. The Dunedin Study: Study Sample eMethods 8. The Dunedin Study: Adverse Childhood Experiences (ACEs) eMethods 9. The Dunedin Study: Mental Health Problems at Age 45 eMethods 10. The Dunedin Study: Physical Health Problems at Age 45 eMethods 11. The Dunedin Study: Clinically Available Childhood Risk Factors eMethods 12. The Dunedin Study: Clinically Available Adult Risk Factors eMethods 13. STROBE Statement—Checklist of Items That Should be Included in Reports of Cohort Studies eTable 1. Analytic Sample Sizes and Prevalence of Health Outcomes by ACE Score eTable 2. Association Between ACEs and Individual Mental Health Problems in the E-Risk and Dunedin Cohorts eTable 3. Association Between ACEs and Individual Physical Health Problems in the E-Risk and Dunedin Cohorts eTable 4. Prediction Accuracy for Health Problems Based on ACE Cut-Off Score of 4+ vs 0-3 ACEs eTable 5. Sensitivity Analysis for Prediction Accuracy in the E-Risk Cohort Testing Potential Bias Arising From Clustered (Twin) Data eTable 6. Association Between ACEs and Health Problems in the E-Risk and Dunedin Cohorts Based on Logistic Regression Models eTable 7. Likelihood Ratios Indexing the Predictive Ability of ACE Scores for Health Problems in the E-Risk and Dunedin Cohorts eFigure 1. Population Representativeness of the E-Risk Study eFigure 2. Prevalence of ACEs in the E-Risk and Dunedin Cohorts in Comparison to the CDC ACEs Study eFigure 3. Attrition Analysis in the Dunedin Study eFigure 4. Prevalence of Mental Health Problems in the E-Risk Cohort and in the Dunedin Cohort According to ACE Score eFigure 5. Prevalence of Ph [file jamapediatr-e205602-s001.pdf]

Supplementary Online Content

Baldwin JR, Caspi A, Meehan AJ, et al. Population vs individual prediction of poor health from results of adverse childhood experiences screening. *JAMA Pediatr.* Published online January 25, 2021. doi:10.1001/jamapediatrics.2020.5602

eMethods 1. Rationale for Including Selected Health Outcomes
eMethods 2. The E-Risk Study: Study Sample
eMethods 3. The E-Risk Study: Adverse Childhood Experiences (ACEs)
eMethods 4. The E-Risk Study: Mental Health Problems at Age 18
eMethods 5. The E-Risk Study: Physical Health Problems at Age 18
eMethods 6. The E-Risk Study: Clinically Available Childhood Risk Factors
eMethods 7. The Dunedin Study: Study Sample
eMethods 8. The Dunedin Study: Adverse Childhood Experiences (ACEs)
eMethods 9. The Dunedin Study: Mental Health Problems at Age 45
eMethods 10. The Dunedin Study: Physical Health Problems at Age 45
eMethods 11. The Dunedin Study: Clinically Available Childhood Risk Factors
eMethods 12. The Dunedin Study: Clinically Available Adult Risk Factors
eMethods 13. STROBE Statement—Checklist of Items That Should be Included in Reports of Cohort Studies
eTable 1. Analytic Sample Sizes and Prevalence of Health Outcomes by ACE Score
eTable 2. Association Between ACEs and Individual Mental Health Problems in the E-Risk and Dunedin Cohorts
eTable 3. Association Between ACEs and Individual Physical Health Problems in the E-Risk and Dunedin Cohorts
eTable 4. Prediction Accuracy for Health Problems Based on ACE Cut-Off Score of 4+ vs 0-3 ACEs
eTable 5. Sensitivity Analysis for Prediction Accuracy in the E-Risk Cohort Testing Potential Bias Arising From Clustered (Twin) Data
eTable 6. Association Between ACEs and Health Problems in the E-Risk and Dunedin Cohorts Based on Logistic Regression Models
eTable 7. Likelihood Ratios Indexing the Predictive Ability of ACE Scores for Health Problems in the E-Risk and Dunedin Cohorts
eFigure 1. Population Representativeness of the E-Risk Study
eFigure 2. Prevalence of ACEs in the E-Risk and Dunedin Cohorts in Comparison to the CDC ACEs Study
eFigure 3. Attrition Analysis in the Dunedin Study
eFigure 4. Prevalence of Mental Health Problems in the E-Risk Cohort and in the Dunedin Cohort According to ACE Score
eFigure 5. Prevalence of Physical Health Problems in the E-Risk Cohort and in the Dunedin Cohort According to ACE Score
eFigure 6. Predictive Accuracy for Individual Mental Health Problems Based on ACE Scores in the E-Risk and Dunedin Cohorts
eFigure 7. Predictive Accuracy for Individual Physical Health Problems Based on ACE Scores in the E-Risk and Dunedin Cohorts
eReferences

This supplementary material has been provided by the authors to give readers additional information about their work.

eMethods 1. Rationale for Including Selected Health Outcomes

The health outcomes examined in this paper include (a) any mental health problem, (b) any physical health problem, and (c) specific mental health problems (depression, anxiety, self-harm, suicide attempt, attention-deficit hyperactivity disorder (ADHD), alcohol dependence, and drug dependence) and (d) specific physical health problems (obesity, high inflammation levels, asthma, sexually transmitted disease (STD), sleep problems, and smoking). We selected these health problems because (1) equivalent outcomes were assessed in the original CDC ACE Study[1], and (2) they were consistently assessed in both the E-Risk and Dunedin cohorts and are relevant in both late adolescence and middle age. We initially focused on general measures of 'any' mental/physical health problem because: (1) ACEs have non-specific associations with a wide range of health outcomes (e.g. 23 outcomes in a meta-analysis)[2], and (2) there is high comorbidity among mental health problems and physical health problems. Subsequently, we tested the sensitivity of the results across individual health outcomes.

The selection of health outcomes was defined a priori in a pre-registration (see: https://sites.google.com/site/moffittcaspiprojects/home/approved_2018/baldwinj_2018a). In addition, ADHD was later added to maximize the number of externalizing mental health problems represented, and asthma and STDs were added to maximize the number of physical health outcomes assessed in both cohorts.

eMethods 2. The E-Risk Study: Study Sample

Participants were members of the Environmental Risk (E-Risk) Longitudinal Twin Study, which tracks the development of a birth cohort of 2232 British children. The sample was drawn from a larger birth register of twins born in England and Wales in 1994-95.[3] Full

details about the sample are reported elsewhere.[4] Briefly, the E-Risk sample was constructed in 1999-2000, when 1,116 families (93% of those eligible) with same-sex 5-year-old twins participated in home-visit assessments. This sample comprised 56% monozygotic (MZ) and 44% dizygotic (DZ) twin pairs; sex was evenly distributed within zygosity (49% male). Seven percent of the study members self-identified as black, Asian, or mixed race. Families were recruited to represent the U.K. population of families with newborns in the 1990s, on the basis of residential location throughout England and Wales and mother's age. Teenaged mothers with twins were over-selected to replace high-risk families who were selectively lost to the register through non-response. Older mothers having twins via assisted reproduction were under-selected to avoid an excess of well-educated older mothers. The study sample represents the full range of socioeconomic conditions in Great Britain, as reflected in the families' distribution on a neighborhood-level socioeconomic index (ACORN [A Classification of Residential Neighbourhoods], developed by CACI Inc. for commercial use in Great Britain):[5] 25.6% of E-Risk families live in "wealthy achiever" neighborhoods compared to 25.3% nationwide; 5.3% vs. 11.6% live in "urban prosperity" neighborhoods; 29.6% vs. 26.9% live in "comfortably off" neighborhoods; 13.4% vs. 13.9% live in "moderate means" neighborhoods; and 26.1% vs. 20.7% live in "hard-pressed" neighborhoods. E-Risk underrepresents "urban prosperity" neighborhoods because such households are likely to be childless. The study sample is also equally distributed across all deciles of the Index of Multiple Deprivation (IMD) 2015, which measures relative levels of deprivation in small areas in England (eFigure 5).

Follow-up home visits were conducted when the children were aged 7 (98% participation), 10 (96%), 12 (96%), and 18 (93%). Home visits at ages 5, 7, 10, and 12 years included assessments with participants as well as their mother (or primary caretaker); the home visit at age 18 included interviews only with the participants. Each twin participant was assessed by a different interviewer. The average age of the twins at the time of the assessment was 18.4 years (SD = 0.36); all interviews were conducted after the 18th birthday. There were no

© 2021 Baldwin JR et al. *JAMA Pediatrics*.

differences between the 2,066 participants who took part at age 18 and those who did not in terms of socioeconomic status (SES) assessed when the cohort was initially defined (χ^2=0.86, p=0.65), age-5 IQ scores (t=0.98, p=0.33), or adverse childhood experiences (χ^2=0.60, p=0.97). Of the 2066 children who participated in the E-Risk Study assessments at age 18, 2,009 (97%) had complete data for adverse childhood experiences (ACEs) and health outcomes at age 18 and were included in the analysis.

The Joint South London and Maudsley and the Institute of Psychiatry Research Ethics Committee approved each phase of the study. Parents gave informed consent and twins gave assent between 5-12 years and then informed consent at age 18.

eMethods 3. The E-Risk Study: Adverse Childhood Experiences (ACEs)

The E-Risk Study assessed five types of child harm (physical abuse, sexual abuse, emotional abuse, emotional neglect, and physical neglect) and five types of household dysfunction (household partner violence, household substance abuse, family mental illness, parental antisocial behaviour, and parental separation) to correspond to the 10 categories of childhood adversity introduced by the CDC Adverse Childhood Experiences Study.[1] These experiences were assessed from records gathered over four home visits from ages 5 to 12 years. These records included the following: structured notes from interviews with parents using the MultiSite Child Development Project interview[6,7] (to assess child harm), the Conflict Tactics Scale[8] (to assess household partner violence), the Young Adult Behavior Checklist[9] (to assess parental antisocial behaviour), and the Family History Screen[10,11] (to assess household substance abuse and family history of mental illness); observations by research workers of the family environment using the Home Observation for Measurement of the Environment[12] (including assessments of parent-child interactions and whether the environment was safe, sanitary and healthy), and information from clinicians whenever the E-Risk Study team made a child-protection referral.

© 2021 Baldwin JR et al. *JAMA Pediatrics*.

Each category of childhood adversity was coded as a binary exposure: physical abuse, emotional abuse, physical neglect, emotional neglect, and sexual abuse were coded if there was evidence of severe exposure;[13] household partner violence was coded if there was evidence that it was repeated; household substance abuse was defined as approximately the top quarter of the proportion of family members with a history of substance use; family mental illness was defined as a report of hospitalization for psychiatric disorder or substance-use problem, or attempted or completed suicide for any of the child's biological mother, father, grandparents, or aunts and uncles; parental antisocial behaviour was coded as the top quarter of the variety of antisocial behaviors of parents, and parental separation was defined as one or both biological parent(s) being absent from the household at some point. Further descriptions of each component ACE measure can be found in Beckley et al.[14]

ACE summary score. We derived an ACE summary score by summing the dichotomised component measures (namely, physical abuse, sexual abuse, emotional abuse and neglect, physical neglect, household partner violence, household substance abuse, family mental illness, parental antisocial behaviour, and parental separation). Participants were excluded if they were missing data for more than one ACE. We truncated the ACE score at 4+ ACEs in line with conventions in research[2,15] and clinical practice.[16] eFigure 7 shows the prevalence of ACEs in E-Risk compared to the Dunedin cohort and CDC ACEs Study.[1]

Binary 4+ ACE cut-off score. We also derived a binary cut-off score indexing whether participants experienced four or more ACEs or not, for sensitivity analyses. As with the ACE summary score, participants were excluded if they were missing data for more than one ACE.

eMethods 4. The E-Risk Study: Mental Health Problems at Age 18

During age 18 interviews, we assessed the presence of major depressive disorder (referred to as depression), generalized anxiety disorder (referred to as anxiety), self-harm, suicide attempt, attention-deficit-hyperactivity disorder (ADHD), alcohol dependence, and drug

© 2021 Baldwin JR et al. *JAMA Pediatrics*.

dependence (comprising dependence on marijuana and/or other drugs) according to Diagnostic and Statistical Manual of Mental Disorders (DSM-5) criteria.[17] The reporting period referred to the previous 12 months, with the exception of self-harm and suicide attempt, in which a 6-year reporting period was used.[18] Furthermore, for the anxiety diagnosis, we did not require the 6-month symptom duration criterion because of the young age of our study sample. Assessments were conducted in face-to-face private interviews using the Diagnostic Interview Schedule.[19]

Any mental health problem. We derived a composite measure reflecting the presence of any mental health problem at age 18 (e.g., depression, anxiety, self-harm, suicide attempt, ADHD, alcohol dependence, or drug dependence). Participants who were missing data for all mental health problems at age 18 were excluded.

eMethods 5. The E-Risk Study: Physical Health Problems at Age 18

Obesity. Body mass index (BMI) at age 18 was calculated by dividing weight in kilograms by height in metres squared; obesity was defined as BMI≥30.

Inflammation. We assessed elevated systematic inflammation through high-sensitivity assays of C-reactive protein (hsCRP). hsCRP was collected in capillary blood on filter paper during a home visit at the age-18 follow-up assessment in 1937 Study members (94% of those who took part in the assessment).[20] Study members' fingers were sterilized with an alcohol swab and incised with a retractable lancet (Bunzel Healthcare, catalogue number 366594). The first blood drop was discarded on tissue. Five subsequent drops of 50ul each were collected on a protein saver card (Fisher Scientific, catalogue number 10531018). The cards were placed in an air-tight drying box containing 2 Maxipax absorbent packets each containing 10 g silica gel and allowed to dry completely overnight. The cards were then moved into Ziplock bags with at least 2 absorbent packets per card and a humidity indicator card and refrigerated. The cards were transported to a central laboratory at the SGDP Centre for storage at 80 C within 2 weeks of collection. The following morning, samples were

shaken on a plate shaker for 30 min. hsCRP was quantified via Sandwich ELISA using the Human C-Reactive Protein ELISA Kit KHA0031 (Life Technologies, UK) according to manufacturers' instructions. The kit has a lower limit of detection <10 pg/mL. Standard deviations were calculated from the samples' duplicates giving a coefficient of variation of 3.3%. Paired collection of dried blood spot and serum in n = 98 Study members was performed to derive a within-study conversion equation for hsCRP levels, where serum CRP value = 6.51 * (blood spot CRP value) + 0.14. We excluded 64 Study members with serum-equivalent CRP >10 mg/L as they were likely to have acute trauma, infections, or pathology.[21] We defined high inflammation levels as hsCRP>3 mg/L, in line with the Centers for Disease Control and Prevention (CDC)/American Heart Association (AHA) definition of high cardiovascular risk.[22]

Asthma. Asthma since age 12 were assessed at age 18 in a private individual interview conducted by trained professionals.[23] Participants were asked "Since age 12, have you been told by a doctor that you have asthma?" Those who responded "yes" were coded as having asthma.

Sexually transmitted diseases (STDs). Participants answered questions about sexual health at age 18 via a private computer-administered questionnaire based on the 1990 British National Survey of Sexual Attitudes and Lifestyles.[24,25] Participants were first asked if they had ever had sexual intercourse, and if so, were subsequently asked whether they had been told by a doctor since age 12 that they had Chlamydia, Genital Warts of Human Papillomavirus, Gonorrhea, genital herpes, viral hepatitis, or any other STDs. Reports of any of these were taken to indicate that the participant had contracted an STD. Participants who had not had sexual intercourse were coded as not having an STD.

Sleep problems. We measured sleep quality at age 18 years using the Pittsburgh Sleep Quality Index (PSQI).[26] The PSQI consists of 18 self-report items relating to individuals' sleep patterns and different forms of sleep impairment in the past month. These questions

© 2021 Baldwin JR et al. *JAMA Pediatrics*.

are used to derive scores for seven different components of sleep (subjective sleep quality, sleep latency, sleep duration, habitual sleep efficiency, sleep disturbances, use of sleep medication and daytime dysfunction), each scored from 0 to 3. These were summed to produce a global score ranging from 0 to 21, with higher scores reflecting worse sleep quality. We defined sleep problems as a score higher than 5 on the PSQI, which has been proposed as a clinical cut-off.[26]

Smoking. Daily cigarette smoking at age 18 was assessed by asking the participant if they had ever smoked a cigarette, followed by if and when they began smoking every day; current daily smokers were participants who endorsed daily smoking within the past year.

Any physical health problem. We derived a composite measure reflecting the presence of any physical health problem at age 18 (e.g., obesity, high inflammation levels, asthma, STD, sleep problems, and smoking). Participants who were missing data for all physical health problems at age 18 were excluded.

eMethods 6. The E-Risk Study: Clinically Available Childhood Risk Factors

To test whether prospectively ascertained ACE scores predicted health problems over and above other clinically available information, we adjusted for health risk factors in childhood that could be readily assessed by clinicians: namely, sex, family socioeconomic status, childhood mental health problems (for analyses on later mental health outcomes) and childhood physical health problems (for analyses on later physical health outcomes). We did not include ethnicity given the low ethnic heterogeneity of the E-Risk sample**.**

Family socioeconomic status. Family socioeconomic status was defined using a standardized composite of parents' income, education and social class ascertained at childhood phases of the study, which loaded significantly onto one latent factor.[27] The population-wide distribution of the resulting factor was divided in tertiles for analyses.

© 2021 Baldwin JR et al. *JAMA Pediatrics.*

Child mental health. At ages 5, 7, 10, and 12 years, parents and teachers completed the Child Behavior Checklist (CBCL) and teacher's report form (TRF), respectively.[28,29] Childhood internalizing problems were assessed through the withdrawn/depressed and somatic subscales, and childhood externalizing problems were assessed through the delinquency and aggression subscales. The total scores for internalizing and externalizing problems (respectively) were standardized and averaged across raters and assessments. We defined poor child mental health as a score of ≥1 standard deviation above the cohort means for internalizing or/and externalizing problems (23.4%; N=518). Participants who were missing data for both internalizing and externalizing problems were excluded.

Childhood physical health. We measured children's physical health from parent reports and clinical ratings of illnesses and health conditions taken at assessments spanning birth to age 12 years. Long-term illnesses since birth were assessed at age 5 through mother's reports using an event history calendar.[30] Asthma was assessed at ages 5 and 10 through collecting information from mothers using an event history calendar.[23] Overweight was assessed at ages 10 and 12 through research workers' ratings of children's weights on a 7-point scale (with 1 being underweight and 7 being overweight).[31] Research worker ratings of children's weight at age 10 were correlated with their ratings at age 12 (r = 0.58). We defined overweight in childhood as a score of 6 or 7 at either age 10 or 12 years. Smoking in the past six months was assessed at age 12 through mother's report on the item "smokes tobacco" on the CBCL. We defined smoking as a response of "sometimes or somewhat true" or "very often true". From this information, we derived an overall measure of poor child physical health indexing the presence of any childhood long-term illness, asthma, overweight, or smoking. Participants who were missing data for all childhood physical health problems were excluded.

© 2021 Baldwin JR et al. *JAMA Pediatrics*.

eMethods 7. The Dunedin Study: Study Sample

Participants are members of the Dunedin Multidisciplinary Health and Development Study, a longitudinal investigation of health and behavior in a complete birth cohort. Study members (*N* = 1,037; 91% of eligible births; 52% male, 48% female) were all individuals born between April 1972 and March 1973 in Dunedin, New Zealand, who were eligible for the longitudinal study based on residence in the province at age 3 and who participated in the first follow-up assessment at age 3. The cohort represents the full range of socioeconomic status in the general population of New Zealand's South Island and, as adults, matches the New Zealand National Health and Nutrition Survey on key adult health indicators (eg, body mass index, smoking, and general practitioner visits) and the New Zealand Census of citizens of the same age on educational attainment. Cohort members were primarily white (93%), which matches the ethnic distribution of the South Island of New Zealand.[32] Assessments were carried out at ages 3, 5, 7, 9, 11, 13, 15, 18, 21, 26, 32, 38, and most recently, 45 years, when 938 of the 997 participants (94.1%) still alive participated. Participants at age 45 years did not differ significantly from other living participants in terms of childhood IQ, childhood SES, or ACEs (eFigure 6).

At each assessment wave, study members are brought to the Dunedin Multidisciplinary Health and Development Research Unit for a full day of interviews and examinations. These data are supplemented by searches of official records and by questionnaires that are mailed, as developmentally appropriate, to parents, teachers, and informants nominated by the study members themselves. The University of Otago Ethics Committee approved each phase of the study.

eMethods 8. The Dunedin Study: Adverse Childhood Experiences (ACEs)

The Dunedin Study assessed five types of child harm (physical abuse, sexual abuse, emotional abuse, emotional neglect, and physical neglect) and five types of household

© 2021 Baldwin JR et al. *JAMA Pediatrics.*

dysfunction (household partner violence, household substance abuse, family mental illness, parental criminality, and parental separation) to correspond to the 10 categories of childhood adversity introduced by the CDC Adverse Childhood Experiences Study.[1] These adversities were assessed both prospectively in childhood and retrospectively in adulthood. eFigure 7 shows the prevalence of prospectively and retrospectively measured ACEs in the Dunedin cohort compared to the E-Risk cohort and CDC ACEs Study.[1]

Prospective ACE measure:

Prospective ACE counts were generated from archival Dunedin Study records gathered during seven biennial assessments carried out from ages 3 to 15 years. The records include the following: social service contacts; structured notes from assessment staff who interviewed Study children and their parents; structured notes from pediatricians and psychometricians who observed mother–child interactions at the research unit; structured notes from nurses who recorded conditions witnessed at home visits; and notes of concern from teachers who were surveyed about the Study children's behavior and performance. Separately, parental criminality was surveyed via postal questionnaire to the parents.

Archival Study data were reviewed in 2015 by four independent raters who were trained on the CDC definitions of ACEs. Individual ACEs were agreed upon by at least three of the four raters 80% of the time. The sole exception was emotional neglect where half the cases were identified by only two raters. Agreement across the full ACE count between the four raters ranged from kappa = .76 to .82, with an average inter-rater agreement kappa of .79. The completeness of archival Dunedin Study records of adversity varied by the type of ACE considered. Some ACEs (notably childhood sexual abuse) will have been underdetected to the extent that these experiences were not actively queried, reflecting assumptions in the 1970s that sexual abuse was exceedingly rare.[33]

ACE summary score. We derived a prospective ACE score by summing the dichotomised component measures (namely, physical abuse, sexual abuse, emotional abuse and neglect,

© 2021 Baldwin JR et al. *JAMA Pediatrics*.

physical neglect, household partner violence, household substance abuse, family mental illness, parental criminality, and parental separation). We truncated the ACE score at 4+ ACEs in line with conventions in research[1,2] and clinical practice.[16]

Binary 4+ ACE cut-off score. We also derived a binary cut-off score indexing whether participants had prospective evidence of four or more ACEs or not (i.e., exposure to 0-3 ACEs), for sensitivity analyses.

Retrospective ACE measure:

ACEs were retrospectively assessed through structured interviews conducted when Dunedin Study participants were adults. Like the original CDC ACE Study,[1] we administered the Childhood Trauma Questionnaire (CTQ),[34] which ascertains physical, sexual, and emotional abuse, physical neglect, and emotional neglect; the CTQ was administered at age 38. Following the CTQ manual, a specific category of harm was present if the Study member had a moderate to severe score. Study members were also interviewed about memories of exposure to family substance abuse, mental illness, and incarceration during childhood via the Family History Screen.[11] Exposure to partner violence was assessed by asking Study participants, 'Did you ever see or hear about your mother/father being hit or hurt by your father/mother/stepfather/stepmother?' We also interviewed participants about parental loss (due to separation, divorce, death, or removal from home).

ACE summary score. We derived a retrospective ACE score by summing the dichotomised component measures (namely, physical abuse, sexual abuse, emotional abuse and neglect, physical neglect, household partner violence, household substance abuse, family mental illness, parental criminality, and parental separation). We truncated the ACE score at 4+ ACEs, in line with conventions in research[1,2] and clinical practice.[16]

Binary 4+ ACE cut-off score. We also derived a binary cut-off score indexing whether participants retrospectively reported four or more ACEs or not (i.e., exposure to 0-3 ACEs), for sensitivity analyses.

© 2021 Baldwin JR et al. *JAMA Pediatrics*.

eMethods 9. The Dunedin Study: Mental Health Problems at Age 45

Clinically trained interviewers conducted private interviews with the study members at 45 years of age using the Diagnostic Interview Schedule (DIS)[19,35] to assess the presence of psychiatric disorders over the previous 12 months. Diagnoses of major depression, generalized anxiety disorder, and ADHD were made according to the symptom and impairment criteria from the DSM-5.[36] Diagnoses of alcohol dependence, marijuana dependence, and drug dependence were made according to criteria from the DSM-IV,[37] and marijuana dependence and drug dependence were combined into one overall drug dependence measure. Self-harm and suicide attempts since age 38 years were also assessed at age 45 during structured interviews about self-harm and suicide. Interviewers differentiated between suicide attempts and nonsuicidal self-harm.

Any mental health problem. We derived a composite measure reflecting the presence of any mental health problem at age 45 (e.g., depression, anxiety, self-harm, suicide attempt, ADHD, alcohol dependence, and drug dependence). Participants who were missing data for all mental health problems at age 45 were excluded.

eMethods 10. The Dunedin Study: Physical Health Problems at Age 45

Obesity. Individuals' height and weight were measured at age 45. Height was measured to the nearest millimeter using a Seca 264 Wireless Stadiometer. Weight was measured to the nearest 0.1 kg using calibrated scales. Individuals were weighed in light clothing. Obesity was defined as a BMI≥30.

Inflammation. Elevated systemic inflammation at age 45 was assessed using a high-sensitivity immunoturbidimetric assay of C-reactive protein (hsCRP) in serum. HsCRP was measured on a Cobas c702 analyzer. Study members with serum-equivalent CRP values >10 mg/L were excluded as they were likely to have acute trauma, infections, or pathology.[21] The Centers for Disease Control and Prevention (CDC)/American Heart Association (AHA)

© 2021 Baldwin JR et al. *JAMA Pediatrics.*

definition of high cardiovascular risk (hsCRP>3 mg/L) was adopted to identify the risk group.[22]

Asthma. Current asthma at age 45 was defined as a self-reported current diagnosis at least one of (1) recurrent wheeze, (2) asthma attack, or (3) asthma medication use in the past year.

Sexually transmitted diseases (STDs). At age 45, Study members were asked whether they had experienced one or more STDs since the previous assessment at age 38.[38] Conditions were identified from a list of the common STDs (chlamydia, non-specific urethritis [NSU], genital warts, herpes, gonorrhoea, trichomoniasis, syphilis), or specified as an “other STI”. Reports of any of these STDs were taken to indicate that the participant had contracted an STD between ages 38 and 45.

Sleep problems. We measured sleep quality at age 45 using the Pittsburgh Sleep Quality Index (PSQI).[26] The PSQI consists of 18 self-report items relating to individuals’ sleep patterns and different forms of sleep impairment in the past month. These questions are used to derive scores for seven different components of sleep (subjective sleep quality, sleep latency, sleep duration, habitual sleep efficiency, sleep disturbances, use of sleep medication and daytime dysfunction), each scored from 0 to 3. These were summed to produce a global score ranging from 0 to 21, with higher scores reflecting worse sleep quality. We defined sleep problems as a score higher than 5 on the PSQI, which has been proposed as a clinical cut-off.[26]

Smoking. Current smoking was defined as smoking at least one cigarette daily for at least 1 month in the previous year based on reports by the participants at age 45.

Any physical health problem. We derived a composite measure reflecting the presence of any physical health problem at age 45 (e.g., obesity, high inflammation levels, asthma, STD,

© 2021 Baldwin JR et al. *JAMA Pediatrics*.

sleep problems, and smoking). Participants who were missing data for all physical health problems at age 45 were excluded.

eMethods 11. The Dunedin Study: Clinically Available Childhood Risk Factors

To test whether prospectively ascertained ACE scores predicted health problems over and above other clinically available information, we adjusted for health risk factors in childhood that could be readily assessed by clinicians: namely, sex, family socioeconomic status, childhood mental health problems (for analyses on later mental health outcomes) and childhood physical health problems (for analyses on later physical health outcomes). We did not include ethnicity given the low ethnic heterogeneity of the Dunedin sample.

Family socioeconomic status. Childhood socioeconomic status (SES) was defined as the average of the highest occupational status level of either parent across study assessments from the Study member's birth through 15 years (1=unskilled labourer; 6=professional), on New Zealand's occupational rating of the 1970s.

Child mental health. At ages 5, 7, 9, and 11, parents and teachers completed the Rutter Child Scale A and B, respectively, and additional items assessing inattention, impulsivity, and hyperactivity. Subscales, based upon factor analysis of all items, have been formed for measuring hyperactivity, antisocial behaviour, and worry-fearfulness.[39] We defined poor child mental health as a score of ≥1 standard deviation above the cohort mean for hyperactivity, antisocial behaviour, or worry-fearfulness (29.6%; N=304). Participants who were missing data for hyperactivity, antisocial behaviour, and worry-fearfulness were excluded.

Child physical health. We measured cohort members' childhood health from a panel of biomarkers and clinical ratings taken at assessments spanning birth to age 11 years.[40] Motor development was assessed at ages 3, 5, 7, and 9 using the Bailey Motor Scales (age 3)[41], McCarthy Motor Scales[42] (age 5) and Basic Motor Ability Test[43] (ages 7 and 9). Children's overall health at ages 3, 5, 7, 9, and 11 years was rated by two Unit staff

© 2021 Baldwin JR et al. *JAMA Pediatrics*.

members based on review of birth records and assessment dossiers including clinical assessments and reports of infections, diseases, injuries, hospitalizations, and other health problems collected from children's mothers during standardized interviews. Ratings were made on a five-point scale (inter-rater agreement=0.85). Body mass index was calculated from height and weight measurements taken at ages 5, 7, 9, and 11 years. In addition, tricep and subscapular skinfold thicknesses were measured at ages 7 and 9 years by trained anthropometrists.[44] (For calculation of the overall measure, tricep and subscapular skinfold thicknesses were averaged to create a single score.) Systolic and diastolic blood pressure were measured at ages 7, 9, and 11 years using a London School of Hygiene and Tropical Medicine blind mercury sphygmomanometer (Cinetronics Ltd., Mildenhall, United Kingdom).[45] Fixed expiratory volume in one second (FEV1) and the ratio of FEV1 to forced vital capacity (FVC) were measured at ages 9 and 11 using a Godart water spirometer.[46] To calculate the childhood health measure, assessments were standardized to have mean=0 and SD=1 within age and sex specific groups. Cross-age scores for each measure were then computed by averaging standardized scores across measurement ages. Reliabilities for measurements are, for girls/boys Motor Ability 0.79/0.73; Clinician Health Rating 0.66/0.68; BMI 0.92/0.93; Tricep Skinfold Thickness 0.85/0.75; Subscapular Skinfold Thickness 0.90/0.85; Systolic Blood Pressure 0.81/0.84; Diastolic Blood Pressure 0.57/0.69; FEV1 0.92/0.96; FEV1/FVC 0.84/0.85. The summary childhood health score was calculated by taking the natural log of the average score across all measures, resulting in a normally distributed childhood health index. We defined poor childhood physical health as a score of ≥ 1 standard deviation above the mean (15.5% of children; N=150).

eMethods 12. The Dunedin Study: Clinically Available Adult Risk Factors

To test whether retrospectively ascertained ACE scores predicted health problems over and above other clinically available information, we adjusted for health risk factors in adulthood that could be readily assessed by clinicians: namely, sex, socioeconomic status, and self-reported health. We included socioeconomic status and self-reported health assessed in

© 2021 Baldwin JR et al. *JAMA Pediatrics.*

adulthood (age 38) in order to reflect information that clinicians could acquire about adult individuals during a retrospective ACE screening assessment.

Socioeconomic status. Socioeconomic status was assigned based on each participant's current occupation at age 38 years. The New Zealand Socioeconomic Index (NZSEI-06) codes each occupation based on its associated education level and income in the NZ Census.[47] Socioeconomic status for 19 unemployed participants was assigned based on their most recent occupation in their thirties, and socioeconomic status for 14 homemakers was imputed from their education following the NZSEI-06 algorithm (score range, 10 [low status]-90 [high status]). The NZSEI-06 scores are further grouped into 6 socioeconomic status groups. Examples of occupations in the 6 groups include medical practitioner (group 6), engineering professional (group 5), database administrator (group 4), personal assistant (group 3), office cashier (group 2), and fish filleter (group 1).

Self-reported health at age 38. Self-rated poor health was measured at age 38 by a 5-point scale in response to the question: 'In general, would you say your health is?' Response options were 'poor', 'fair', 'good', 'very good', or 'excellent'.[48]

© 2021 Baldwin JR et al. *JAMA Pediatrics*.

eMethods 13. STROBE Statement—Checklist of Items That Should be Included in Reports of Cohort Studies

	Item No	Recommendation	Page No
**Title and abstract**	1	(*a*) Indicate the study's design with a commonly used term in the title or the abstract	1
		(*b*) Provide in the abstract an informative and balanced summary of what was done and what was found	1
**Introduction**			
Background/rationale	2	Explain the scientific background and rationale for the investigation being reported	2
Objectives	3	State specific objectives, including any prespecified hypotheses	2
**Methods**			
Study design	4	Present key elements of study design early in the paper	2-3
Setting	5	Describe the setting, locations, and relevant dates, including periods of recruitment, exposure, follow-up, and data collection	2-3, eMethods 2 and eMethods 7 in the Supplement
Participants	6	(*a*) Give the eligibility criteria, and the sources and methods of selection of participants. Describe methods of follow-up	eMethods 2 and eMethods 7 in the Supplement
		(*b*) For matched studies, give matching criteria and number of exposed and unexposed	
Variables	7	Clearly define all outcomes, exposures, predictors, potential confounders, and effect modifiers. Give diagnostic criteria, if applicable	3-4, eMethods 3-6, eMethods 8-12 in the Supplement
Data sources/ measurement	8*	For each variable of interest, give sources of data and details of methods of assessment (measurement). Describe comparability of assessment methods if there is more than one group	eMethods 3-6 and eMethods 8-12 in the Supplement
Bias	9	Describe any efforts to address potential sources of bias	4
Study size	10	Explain how the study size was arrived at	eTable 1 in the Supplement
Quantitative variables	11	Explain how quantitative variables were handled in the analyses. If applicable, describe which groupings were chosen and why	4, eMethods 3-6, eMethods 8-12
Statistical methods	12	(*a*) Describe all statistical methods, including those used to control for confounding	4
		(*b*) Describe any methods used to examine subgroups and interactions	NA
		(*c*) Explain how missing data were addressed	eMethods 3-6, eMethods 9-11, eTable 1 in the Supplement
		(*d*) If applicable, explain how loss to follow-up was addressed	NA

© 2021 Baldwin JR et al. *JAMA Pediatrics.*

	Item No	Recommendation	Page No
		(*e*) Describe any sensitivity analyses	4
**Results**			
Participants	13*	(a) Report numbers of individuals at each stage of study—eg numbers potentially eligible, examined for eligibility, confirmed eligible, included in the study, completing follow-up, and analysed	eMethods 2, eMethods 7
		(b) Give reasons for non-participation at each stage	eMethods 2, eMethods 7
		(c) Consider use of a flow diagram	NA
Descriptive data	14*	(a) Give characteristics of study participants (eg demographic, clinical, social) and information on exposures and potential confounders	4, eMethods 2, eMethods 7, eTable 1
		(b) Indicate number of participants with missing data for each variable of interest	eTable 1
		(c) Summarise follow-up time (eg, average and total amount)	Figure 1, eMethods 2, eMethods 7
Outcome data	15*	Report numbers of outcome events or summary measures over time	eTable 1
Main results	16	(*a*) Give unadjusted estimates and, if applicable, confounder-adjusted estimates and their precision (eg, 95% confidence interval). Make clear which confounders were adjusted for and why they were included	Table 1, Figure 3, eTables 2-3 in the Supplement
		(*b*) Report category boundaries when continuous variables were categorized	NA
		(*c*) If relevant, consider translating estimates of relative risk into absolute risk for a meaningful time period	NA
Other analyses	17	Report other analyses done—eg analyses of subgroups and interactions, and sensitivity analyses	eTables 4-5 in the Supplement
Discussion			
Key results	18	Summarise key results with reference to study objectives	7
Limitations	19	Discuss limitations of the study, taking into account sources of potential bias or imprecision. Discuss both direction and magnitude of any potential bias	7
Interpretation	20	Give a cautious overall interpretation of results considering objectives, limitations, multiplicity of analyses, results from similar studies, and other relevant evidence	7
Generalisability	21	Discuss the generalisability (external validity) of the study results	7
Other information			
Funding	22	Give the source of funding and the role of the funders for the present study and, if applicable, for the original study on which the present article is based	8

© 2021 Baldwin JR et al. *JAMA Pediatrics*.

eTable 1. Analytic Sample Sizes and Prevalence of Health Outcomes by ACE Score

	E-Risk (prospective measure)			Dunedin (prospective measure)			Dunedin (retrospective measure)		
	**ACE score**	**Sample size (N)**	**Prev (%)**	**ACE score**	**Sample size (N)**	**Prev (%)**	**ACE score**	**Sample size (N)**	**Prev (%)**
**Mental health outcomes**									
Any mental health problem	0	659	32.78	0	381	24.41	0	305	18.36
	1	524	36.64	1	298	27.85	1	224	29.91
	2	352	38.92	2	121	34.71	2	118	29.66
	3	215	45.58	3	62	45.16	3	88	31.82
	4+	259	56.37	4+	56	41.07	4+	120	47.50
	Total	2009	39.27	Total	918	29.30	Total	855	28.42
Depression	0	657	16.59	0	381	13.65	0	305	9.84
	1	524	17.56	1	298	14.43	1	224	15.18
	2	351	18.52	2	121	16.53	2	118	13.56
	3	215	22.79	3	62	19.35	3	88	15.91
	4+	259	34.36	4+	56	32.14	4+	120	30.83
	Total	2006	20.14	Total	918	15.80	Total	855	15.32
Anxiety	0	658	6.23	0	381	5.25	0	305	3.61
	1	522	6.70	1	297	5.72	1	224	7.59
	2	351	6.84	2	121	8.26	2	118	4.24
	3	214	7.48	3	62	9.68	3	87	4.60
	4+	258	12.40	4+	56	5.36	4+	120	11.67
	Total	2003	7.39	Total	917	6.11	Total	854	5.97
Self-harm	0	658	11.09	0	380	2.63	0	304	1.32
	1	524	10.69	1	298	3.02	1	224	2.23
	2	352	12.78	2	121	2.48	2	118	3.39
	3	215	17.21	3	62	0.00	3	87	4.60
	4+	258	23.26	4+	54	9.26	4+	120	5.83
	Total	2007	13.50	Total	915	2.95	Total	853	2.81
Suicide attempt	0	658	2.28	0	380	1.05	0	304	0.33
	1	524	2.67	1	298	1.01	1	224	0.89
	2	351	3.99	2	121	2.48	2	118	1.69
	3	215	6.98	3	62	1.61	3	87	1.15
	4+	258	6.98	4+	54	3.70	4+	120	4.17
	Total	2006	3.79	Total	915	1.42	Total	853	1.29
ADHD	0	657	5.18	0	378	3.17	0	302	2.32
	1	523	6.88	1	296	2.36	1	224	2.23
	2	352	8.81	2	121	6.61	2	117	3.42
	3	215	10.70	3	61	8.20	3	86	5.81
	4+	258	13.18	4+	55	9.09	4+	119	9.24
	Total	2005	7.88	Total	911	4.06	Total	848	3.77
Alcohol dependence	0	658	9.42	0	381	8.40	0	305	8.52
	1	522	13.03	1	298	12.75	1	224	11.16
	2	352	13.35	2	121	12.40	2	118	13.56
	3	215	13.95	3	62	20.97	3	88	15.91
	4+	259	17.76	4+	56	10.71	4+	120	13.33
	Total	2006	12.61	Total	918	11.33	Total	855	11.35
Drug dependence	0	659	2.28	0	381	3.94	0	305	2.62
	1	524	3.63	1	298	2.68	1	224	3.12
	2	352	4.26	2	121	5.79	2	118	4.24
	3	215	7.44	3	62	16.13	3	88	2.27
	4+	259	10.42	4+	56	7.14	4+	120	12.50
	Total	2009	4.58	Total	918	4.79	Total	855	4.33

© 2021 Baldwin JR et al. *JAMA Pediatrics.*

	E-Risk (prospective measure)			Dunedin (prospective measure)			Dunedin (retrospective measure)		
	**ACE score**	**Sample size (N)**	**Prev (%)**	**ACE score**	**Sample size (N)**	**Prev (%)**	**ACE score**	**Sample size (N)**	**Prev (%)**
**Physical health outcomes**									
Any physical health problem	0	659	54.63	0	367	76.84	0	306	73.86
	1	524	61.26	1	280	77.50	1	226	77.88
	2	352	71.02	2	111	82.88	2	120	78.33
	3	215	71.63	3	60	85.00	3	88	95.45
	4+	259	77.99	4+	54	92.59	4+	119	84.03
	Total	2009	64.06	Total	872	79.36	Total	859	79.16
Obesity	0	650	6.00	0	366	26.78	0	306	28.10
	1	508	8.46	1	277	36.82	1	222	31.53
	2	346	13.29	2	110	38.18	2	117	34.19
	3	213	12.21	3	60	38.33	3	88	46.59
	4+	252	9.13	4+	53	45.28	4+	118	38.14
	Total	1969	8.99	Total	866	33.37	Total	851	33.14
High CRP	0	591	8.80	0	337	17.21	0	283	18.73
	1	476	10.71	1	246	20.73	1	207	19.81
	2	322	14.29	2	107	23.36	2	110	16.36
	3	195	12.31	3	53	22.64	3	81	25.93
	4+	239	12.97	4+	46	30.43	4+	99	23.23
	Total	1823	11.19	Total	789	20.28	Total	780	20.00
Asthma	0	659	13.05	0	367	15.53	0	304	14.14
	1	523	14.34	1	276	18.12	1	224	16.96
	2	352	15.91	2	110	18.18	2	120	22.50
	3	215	17.21	3	59	16.95	3	88	22.73
	4+	259	17.37	4+	54	25.93	4+	117	17.95
	Total	2008	14.89	Total	866	17.44	Total	853	17.47
STDs	0	647	1.55	0	343	4.08	0	284	4.93
	1	515	2.33	1	258	5.43	1	211	6.64
	2	350	3.43	2	101	7.92	2	109	6.42
	3	211	4.27	3	56	8.93	3	83	8.43
	4+	253	4.74	4+	49	8.16	4+	112	4.46
	Total	1976	2.78	Total	807	5.58	Total	799	5.88
Sleep problems	0	659	35.66	0	361	53.19	0	302	48.68
	1	523	37.28	1	276	52.17	1	221	56.11
	2	352	44.60	2	109	52.29	2	119	47.06
	3	215	37.21	3	57	50.88	3	84	61.90
	4+	259	51.74	4+	52	63.46	4+	116	58.62
	Total	2008	39.89	Total	855	53.22	Total	842	53.09
Smoking	0	658	13.83	0	365	13.42	0	306	11.76
	1	523	16.83	1	277	20.58	1	224	22.77
	2	352	22.16	2	111	24.32	2	119	13.45
	3	215	38.14	3	60	38.33	3	87	31.03
	4+	259	42.08	4+	54	51.85	4+	118	37.29
	Total	2007	22.32	Total	867	21.22	Total	854	20.37

Abbreviations: Prev = prevalence; ADHD = attention deficit hyperactivity disorder; CRP = C-reactive protein; STD = sexually transmitted disease. The sample size for each health outcome includes individuals with complete data for ACEs, the health outcome, and all covariates (e.g., sex, SES, and prior health) based on analyses testing forecasting and incremental prediction by ACEs

© 2021 Baldwin JR et al. *JAMA Pediatrics*.

eTable 2. Association Between ACEs and Individual Mental Health Problems in the E-Risk and Dunedin Cohorts

	**Model 1 (unadjusted)**	**Model 2 (adjusted for sex)**	**Model 3 (adjusted for SES at ACE assessment)**	**Model 4 (adjusted for health at ACE assessment)**	**Model 5 (adjusted for all risk factors)**
**A: E-Risk cohort (age 18) – prospective ACE measure**					
Depression (N=2006)	1.19 (1.12-1.26)	1.19 (1.13-1.27)	1.17 (1.09-1.25)	1.18 (1.10-1.25)	1.16 (1.09-1.24)
Anxiety (N=2003)	1.16 (1.04-1.30)	1.18 (1.05-1.31)	1.18 (1.05-1.33)	1.14 (1.01-1.28)	1.16 (1.03-1.30)
Self-harm (N=2007)	1.21 (1.12-1.31)	1.22 (1.13-1.32)	1.18 (1.08-1.28)	1.17 (1.08-1.27)	1.15 (1.05-1.25)
Suicide attempt (N=2006)	1.37 (1.18-1.59)	1.37 (1.18-1.59)	1.31 (1.12-1.52)	1.26 (1.08-1.48)	1.23 (1.05-1.44)
ADHD (N=2005)	1.26 (1.14-1.39)	1.26 (1.14-1.39)	1.22 (1.10-1.36)	1.18 (1.05-1.31)	1.16 (1.03-1.30)
Alcohol dependence (N=2006)	1.15 (1.06-1.24)	1.14 (1.05-1.24)	1.18 (1.08-1.28)	1.13 (1.04-1.23)	1.16 (1.06-1.27)
Drug dependence (N=2009)	1.46 (1.27-1.67)	1.45 (1.26-1.66)	1.32 (1.14-1.53)	1.37 (1.19-1.58)	1.26 (1.08-1.48)
**B: Dunedin cohort (age 45) – prospective ACE measure**					
Depression (N=918)	1.20 (1.07-1.35)	1.20 (1.07-1.35)	1.20 (1.06-1.36)	1.20 (1.06-1.35)	1.20 (1.05-1.35)
Anxiety (N=917)	1.12 (0.90-1.36)	1.11 (0.90-1.36)	1.15 (0.92-1.41)	1.14 (0.92-1.40)	1.16 (0.93-1.44)
Self-harm (N=915)	1.19 (0.88-1.58)	1.19 (0.88-1.56)	1.24 (0.90-1.66)	1.18 (0.86-1.58)	1.22 (0.88-1.65)
Suicide attempt (N=915)	1.37 (0.90-2.01)	1.37 (0.90-2.01)	1.20 (0.78-1.79)	1.38 (0.90-2.06)	1.22 (0.78-1.84)
ADHD (N=911)	1.39 (1.09-1.73)	1.39 (1.09-1.74)	1.45 (1.13-1.84)	1.30 (1.02-1.64)	1.36 (1.06-1.74)
Alcohol dependence (N=918)	1.16 (1.00-1.33)	1.16 (1.01-1.34)	1.19 (1.03-1.38)	1.15 (0.99-1.32)	1.19 (1.02-1.38)
Drug dependence (N=918)	1.37 (1.10-1.68)	1.37 (1.10-1.68)	1.30 (1.04-1.62)	1.30 (1.04-1.61)	1.25 (0.99-1.57)
**C: Dunedin cohort (age 45) – retrospective ACE measure**					
Depression (N=855)	1.28 (1.16-1.42)	1.28 (1.15-1.42)	1.26 (1.13-1.40)	1.26 (1.14-1.41)	1.23 (1.10-1.37)
Anxiety (N=854)	1.23 (1.03-1.47)	1.22 (1.02-1.45)	1.23 (1.03-1.48)	1.24 (1.03-1.48)	1.22 (1.01-1.46)
Self-harm (N=853)	1.43 (1.11-1.87)	1.41 (1.09-1.82)	1.38 (1.06-1.81)	1.40 (1.08-1.83)	1.31 (1.00-1.72)
Suicide attempt (N=853)	1.74 (1.18-2.70)	1.73 (1.17-2.69)	1.58 (1.12-2.31)	1.63 (1.16-2.34)	1.49 (1.07-2.13)
ADHD (N=848)	1.46 (1.17-1.84)	1.47 (1.17-1.85)	1.40 (1.11-1.78)	1.49 (1.18-1.88)	1.44 (1.13-1.85)
Alcohol dependence (N=855)	1.14 (1.00-1.29)	1.16 (1.02-1.31)	1.12 (0.98-1.27)	1.12 (0.99-1.28)	1.14 (1.00-1.30)
Drug dependence (N=855)	1.46 (1.18-1.80)	1.47 (1.19-1.82)	1.35 (1.09-1.67)	1.44 (1.16-1.79)	1.36 (1.10-1.70)

Abbreviations: ACE = adverse childhood experiences; ADHD = attention deficit hyperactivity disorder; SES = socioeconomic status.
Note: Results are presented as Relative Risks and 95% confidence intervals for health problems per additional ACE experienced. We controlled for covariates measured at the time of ACE assessment to reflect information clinicians would have access to at the time of ACE screening. Specifically, analyses using prospective ACE measures adjusted for risk factors measured in childhood (e.g., family socioeconomic disadvantage; child mental health problems) whereas analyses using the retrospective ACE measure adjusted for risk factors in adulthood (e.g., socioeconomic disadvantage at age 38, self-reported health problems at age 38). We adjusted for sex in analyses based on both prospective and retrospective ACE measures.

© 2021 Baldwin JR et al. *JAMA Pediatrics*.

eTable 3. Association Between ACEs and Individual Physical Health Problems in the E-Risk and Dunedin Cohorts

	Model 1 (unadjusted)	Model 2 (adjusted for sex)	Model 3 (adjusted for SES at ACE assessment)	Model 4 (adjusted for health at ACE assessment)	Model 5 (adjusted for all risk factors)
**A: E-Risk cohort (age 18) – prospective ACE measure**					
Obesity (N=1969)	1.15 (1.05-1.26)	1.16 (1.06-1.26)	1.06 (0.96-1.17)	1.08 (0.99-1.18)	1.03 (0.94-1.13)
High CRP (N=1823)	1.11 (1.02-1.21)	1.12 (1.03-1.22)	1.05 (0.96-1.16)	1.08 (0.99-1.18)	1.06 (0.97-1.16)
Asthma (N=2008)	1.08 (1.00-1.16)	1.08 (1.00-1.16)	1.05 (0.97-1.13)	1.01 (0.94-1.08)	1.00 (0.93-1.08)
STDs (N=1976)	1.32 (1.11-1.57)	1.33 (1.13-1.57)	1.19 (0.99-1.43)	1.31 (1.11-1.55)	1.20 (1.01-1.43)
Sleep problems (N=2008)	1.08 (1.04-1.12)	1.09 (1.05-1.13)	1.07 (1.03-1.12)	1.08 (1.04-1.12)	1.08 (1.03-1.12)
Smoking (N=2007)	1.35 (1.28-1.42)	1.35 (1.28-1.42)	1.24 (1.17-1.32)	1.34 (1.26-1.41)	1.23 (1.16-1.31)
**B: Dunedin cohort (age 45) – prospective ACE measure**					
Obesity (N=866)	1.13 (1.05-1.22)	1.13 (1.05-1.22)	1.10 (1.02-1.19)	1.12 (1.04-1.21)	1.09 (1.01-1.18)
High CRP (N=789)	1.14 (1.02-1.27)	1.13 (1.01-1.26)	1.11 (0.99-1.25)	1.13 (1.01-1.25)	1.10 (0.98-1.23)
Asthma (N=866)	1.10 (0.98-1.23)	1.10 (0.97-1.23)	1.09 (0.96-1.23)	1.08 (0.96-1.21)	1.08 (0.95-1.22)
STDs (N=807)	1.24 (0.99-1.52)	1.24 (0.99-1.52)	1.24 (0.98-1.55)	1.24 (0.99-1.52)	1.24 (0.98-1.54)
Sleep problems (N=855)	1.02 (0.97-1.08)	1.02 (0.97-1.07)	1.02 (0.96-1.08)	1.02 (0.96-1.07)	1.02 (0.96-1.07)
Smoking (N=867)	1.39 (1.27-1.52)	1.39 (1.27-1.52)	1.33 (1.21-1.47)	1.40 (1.27-1.53)	1.34 (1.21-1.47)
**C: Dunedin cohort (age 45) – retrospective ACE measure**					
Obesity (N=851)	1.10 (1.03-1.18)	1.10 (1.03-1.18)	1.09 (1.02-1.16)	1.08 (1.01-1.15)	1.07 (1.00-1.14)
High CRP (N=780)	1.06 (0.96-1.17)	1.06 (0.95-1.16)	1.06 (0.95-1.17)	1.05 (0.95-1.16)	1.03 (0.93-1.14)
Asthma (N=853)	1.09 (0.99-1.21)	1.09 (0.98-1.20)	1.09 (0.98-1.21)	1.07 (0.96-1.18)	1.06 (0.95-1.17)
STDs (N=799)	1.03 (0.84-1.24)	1.03 (0.84-1.25)	1.04 (0.85-1.26)	1.01 (0.82-1.23)	1.02 (0.83-1.24)
Sleep problems (N=842)	1.05 (1.00-1.09)	1.04 (1.00-1.09)	1.04 (0.99-1.09)	1.03 (0.98-1.08)	1.02 (0.98-1.07)
Smoking (N=854)	1.29 (1.18-1.41)	1.30 (1.19-1.42)	1.19 (1.09-1.30)	1.23 (1.12-1.34)	1.16 (1.06-1.27)

Abbreviations: ACE = adverse childhood experiences; CRP = C-reactive protein; SES = socioeconomic status; STDs = sexually transmitted diseases. Note: Results are presented as Relative Risks and 95% confidence intervals per additional ACE experienced. We controlled for covariates measured at the time of ACE assessment to reflect information clinicians would have access to at the time of ACE screening. Specifically, analyses using prospective ACE measures adjusted for risk factors measured in childhood (e.g., family socioeconomic disadvantage; child mental health problems) whereas analyses using the retrospective ACE measure adjusted for risk factors in adulthood (e.g., socioeconomic disadvantage at age 38, self-reported health problems at age 38). We adjusted for gender in analyses based on both prospective and retrospective ACE measures.

© 2021 Baldwin JR et al. *JAMA Pediatrics.*

eTable 4. Prediction Accuracy for Health Problems Based on ACE Cut-Off Score of 4+ vs 0-3 ACEs

	A: E-Risk (prospective measure)		B: Dunedin (prospective measure)		C: Dunedin (retrospective measure)	
	**AUC**	**95% CI**	**AUC**	**95% CI**	**AUC**	**95% CI**
**Mental health outcomes**						
Any mental health problem	0.55	0.53-0.56	0.52	0.50-0.54	0.58	0.55-0.60
Depression	0.56	0.54-0.58	0.54	0.51-0.57	0.59	0.55-0.63
Anxiety	0.55	0.51-0.58	0.50	0.46-0.53	0.57	0.51-0.63
Self-harm	0.55	0.53-0.58	0.56	0.49-0.64	0.57	0.49-0.66
Suicide attempt	0.56	0.51-0.60	0.55	0.45-0.65	0.68	0.53-0.82
ADHD	0.55	0.51-0.58	0.55	0.49-0.61	0.61	0.54-0.69
Alcohol dependence	0.53	0.51-0.56	0.50	0.47-0.52	0.51	0.47-0.55
Drug dependence	0.59	0.54-0.63	0.52	0.47-0.56	0.65	0.58-0.73
**Physical health outcomes**						
Any physical health problem	0.54	0.53-0.55	0.53	0.51-0.54	0.53	0.50-0.55
Obesity	0.50	0.48-0.53	0.52	0.50-0.54	0.52	0.49-0.54
High CRP	0.51	0.49-0.54	0.52	0.50-0.54	0.52	0.49-0.55
Asthma	0.51	0.49-0.53	0.52	0.50-0.55	0.51	0.48-0.54
STDs	0.55	0.49-0.60	0.51	0.47-0.55	0.48	0.44-0.53
Sleep problems	0.53	0.52-0.55	0.51	0.50-0.53	0.52	0.49-0.54
Smoking	0.57	0.55-0.59	0.56	0.53-0.59	0.59	0.55-0.62

Abbreviations: AUC = area under the curve; ADHD = attention deficit hyperactivity disorder; CRP = C-reactive protein; STDs = sexually transmitted diseases. Note: The table shows AUC values for ROC curve analyses testing the discriminative ability of ACE scores (4+ ACEs versus 0-3 ACEs) in the prediction of health outcomes.

© 2021 Baldwin JR et al. *JAMA Pediatrics*.

eTable 5. Sensitivity Analysis for Prediction Accuracy in the E-Risk Cohort Testing Potential Bias Arising From Clustered (Twin) Data

		B: AUC in subsamples comprising 1 twin per pair (n=1,116)										
	A: AUC in sample comprising both twins per pair (n=2,232)	1	2	3	4	5	6	7	8	9	10	Average
**Mental health outcomes**												
Any mental health problem	0.58	0.60	0.58	0.59	0.58	0.60	0.59	0.57	0.57	0.59	0.58	0.59
Depression	0.58	0.62	0.59	0.57	0.59	0.61	0.57	0.59	0.58	0.61	0.57	0.59
Anxiety	0.56	0.56	0.60	0.60	0.56	0.54	0.55	0.57	0.54	0.54	0.57	0.56
Self-harm	0.58	0.60	0.60	0.59	0.59	0.60	0.56	0.57	0.60	0.60	0.57	0.59
Suicide attempt	0.63	0.67	0.69	0.64	0.57	0.63	0.61	0.58	0.64	0.69	0.59	0.63
ADHD	0.60	0.63	0.63	0.64	0.61	0.58	0.64	0.61	0.55	0.59	0.60	0.61
Alcohol dependence	0.56	0.56	0.57	0.58	0.58	0.60	0.57	0.56	0.57	0.54	0.52	0.56
Drug dependence	0.66	0.63	0.66	0.62	0.62	0.65	0.65	0.65	0.67	0.67	0.66	0.65
**Physical health outcomes**												
Any physical health problem	0.60	0.60	0.61	0.61	0.60	0.59	0.60	0.61	0.60	0.60	0.61	0.60
Obesity	0.57	0.60	0.59	0.58	0.54	0.57	0.58	0.57	0.56	0.59	0.56	0.57
High CRP	0.55	0.51	0.56	0.54	0.54	0.52	0.56	0.56	0.54	0.53	0.54	0.54
Asthma	0.54	0.55	0.53	0.53	0.53	0.53	0.53	0.53	0.54	0.53	0.54	0.54
STDs	0.62	0.62	0.63	0.68	0.63	0.67	0.62	0.65	0.59	0.57	0.64	0.63
Sleep problems	0.55	0.55	0.57	0.57	0.56	0.55	0.53	0.54	0.55	0.57	0.54	0.55
Smoking	0.65	0.65	0.66	0.65	0.65	0.65	0.66	0.64	0.64	0.65	0.65	0.65

Abbreviations: ADHD = attention deficit hyperactivity disorder; CRP = C-reactive protein; STDs = sexually transmitted disease. Note: The table shows area under the curve (AUC) values for ROC curve analyses testing the discriminative ability of ACE scores in the prediction of health outcomes. Panel A shows the results for the full sample comprising both twins per pair, while Panel B shows the results for 10 subsamples consisting of only one randomly selected twin per pair. The average prediction performance was similar to the results of the full sample (Panel A), indicating that using twins has not biased our results. The exact sample size for each analysis varied but was drawn from the overall sample of N=2,232 or the subsamples of N=1,116.

© 2021 Baldwin JR et al. *JAMA Pediatrics*.

eTable 6. Association Between ACEs and Health Problems in the E-Risk and Dunedin Cohorts Based on Logistic Regression Models

	**Model 1 (unadjusted)**	**Model 2 (adjusted for sex)**	**Model 3 (adjusted for SES at ACE assessment)**	**Model 4 (adjusted for health at ACE assessment)**	**Model 5 (adjusted for all risk factors)**
**A: E-Risk cohort (age 18) – prospective ACE measure**					
Any mental health problem (N=2009)	1.25 (1.17-1.33)	1.25 (1.17-1.34)	1.22 (1.14-1.31)	1.20 (1.12-1.28)	1.19 (1.10-1.27)
Any physical health problem (N=2009)	1.32 (1.23-1.41)	1.33 (1.24-1.43)	1.23 (1.15-1.33)	1.28 (1.19-1.38)	1.23 (1.14-1.32)
**B: Dunedin cohort (age 45) – prospective ACE measure**					
Any mental health problem (N=918)	1.27 (1.13-1.43)	1.27 (1.13-1.43)	1.26 (1.12-1.43)	1.24 (1.10-1.40)	1.24 (1.09-1.40)
Any physical health problem (N=872)	1.26 (1.08-1.48)	1.26 (1.08-1.48)	1.20 (1.03-1.42)	1.26 (1.08-1.48)	1.20 (1.02-1.42)
**C: Dunedin cohort (age 45) – retrospective ACE measure**					
Any mental health problem (N=855)	1.35 (1.22-1.50)	1.35 (1.22-1.50)	1.31 (1.18-1.46)	1.32 (1.19-1.47)	1.29 (1.16-1.44)
Any physical health problem (N=859)	1.27 (1.12-1.44)	1.27 (1.12-1.45)	1.22 (1.07-1.39)	1.19 (1.05-1.37)	1.16 (1.02-1.33)

Note. Results are presented as odds ratios and 95% confidence intervals for health problems per additional ACE experienced. We controlled for covariates measured at the time of ACE assessment to reflect information clinicians would have access to at the time of ACE screening; analyses using prospective ACE measures adjusted for risk factors measured in childhood (e.g., family socioeconomic disadvantage; child mental health problems) whereas analyses using the retrospective ACE measure adjusted for risk factors in adulthood (e.g., socioeconomic disadvantage at age 38, self-reported health problems at age 38). We adjusted for sex in analyses based on both prospective and retrospective ACE measures. The sample size for each outcome includes individuals with complete data for ACEs, the health outcome, and all covariates (e.g., sex, SES, and prior health measures).

© 2021 Baldwin JR et al. *JAMA Pediatrics*.

eTable 7. Likelihood Ratios Indexing the Predictive Ability of ACE Scores for Health Problems in the E-Risk and Dunedin Cohorts

	A: E-Risk (prospective measure)				B: Dunedin (prospective measure)				C: Dunedin (retrospective measure)			
	NLR	95% CI	PLR	95% CI	NLR	95% CI	PLR	95% CI	NLR	95% CI	PLR	95% CI
**Mental health outcomes**												
Any mental health problem	0.85	0.81-0.90	1.64	1.40-1.92	0.84	0.76-0.93	1.55	1.25-1.92	0.73	0.64-0.83	1.53	1.31-1.80
Depression	0.83	0.77-0.90	1.63	1.38-1.92	0.86	0.76-0.98	1.43	1.11-1.84	0.76	0.66-0.87	1.86	1.47-2.35
Anxiety	0.88	0.78-0.98	1.42	1.11-1.82	0.89	0.73-1.08	1.32	0.90-1.94	0.83	0.70-0.98	2.01	1.29-3.14
Self-harm	0.82	0.75-0.90	1.65	1.38-1.98	0.86	0.72-1.03	3.31	1.44-7.64	0.54	0.32-0.92	1.75	1.32-2.31
Suicide attempt	0.64	0.48-0.85	1.54	1.28-1.85	0.72	0.44-1.20	1.80	0.99-3.27	0.41	0.15-1.08	1.95	1.39-2.73
ADHD	0.74	0.62-0.88	1.40	1.20-1.62	0.67	0.48-0.92	2.00	1.43-2.80	0.66	0.48-0.91	2.07	1.48-2.91
Alcohol dependence	0.72	0.57-0.90	1.14	1.06-1.24	0.72	0.53-0.97	1.21	1.05-1.40	0.83	0.68-1.01	1.28	1.03-1.59
Drug dependence	0.69	0.57-0.83	2.08	1.65-2.62	0.70	0.52-0.93	1.91	1.37-2.65	0.65	0.50-0.84	3.24	2.23-4.72
**Physical health outcomes**												
Any physical health problem	0.76	0.71-0.82	1.54	1.36-1.75	0.86	0.79-0.93	1.72	1.23-2.40	0.82	0.77-0.88	2.17	1.48-3.19
Obesity	0.77	0.66-0.91	1.34	1.16-1.56	0.73	0.61-0.87	1.23	1.10-1.37	0.85	0.75-0.95	1.28	1.09-1.50
High CRP	0.85	0.74-0.98	1.22	1.05-1.42	0.81	0.65-1.01	1.15	1.01-1.31	0.92	0.83-1.02	1.27	0.97-1.67
Asthma	0.90	0.81-1.01	1.15	1.00-1.31	0.81	0.65-1.01	1.15	1.01-1.30	0.85	0.73-0.99	1.25	1.03-1.51
STDs	0.67	0.49-0.93	1.48	1.18-1.84	0.86	0.69-1.06	1.43	0.97-2.10	0.91	0.60-1.38	1.05	0.86-1.28
Sleep problems	0.86	0.80-0.93	1.23	1.11-1.36	0.97	0.94-1.00	1.67	0.97-2.87	0.91	0.84-0.98	1.35	1.07-1.70
Smoking	0.70	0.64-0.76	2.35	2.02-2.73	0.72	0.63-0.81	2.06	1.66-2.54	0.70	0.61-0.79	2.22	1.79-2.75

Abbreviations: NLR = negative likelihood ratio; PLR = positive likelihood ratio; CI = confidence interval; ADHD = attention deficit hyperactivity disorder; CRP = C-reactive protein; STDs = sexually transmitted diseases. Note: The table shows positive and negative likelihood ratios testing the discriminative ability of ACE scores in the prediction of health outcomes.

© 2021 Baldwin JR et al. *JAMA Pediatrics*.

eFigure 1. Population Representativeness of the E-Risk Study

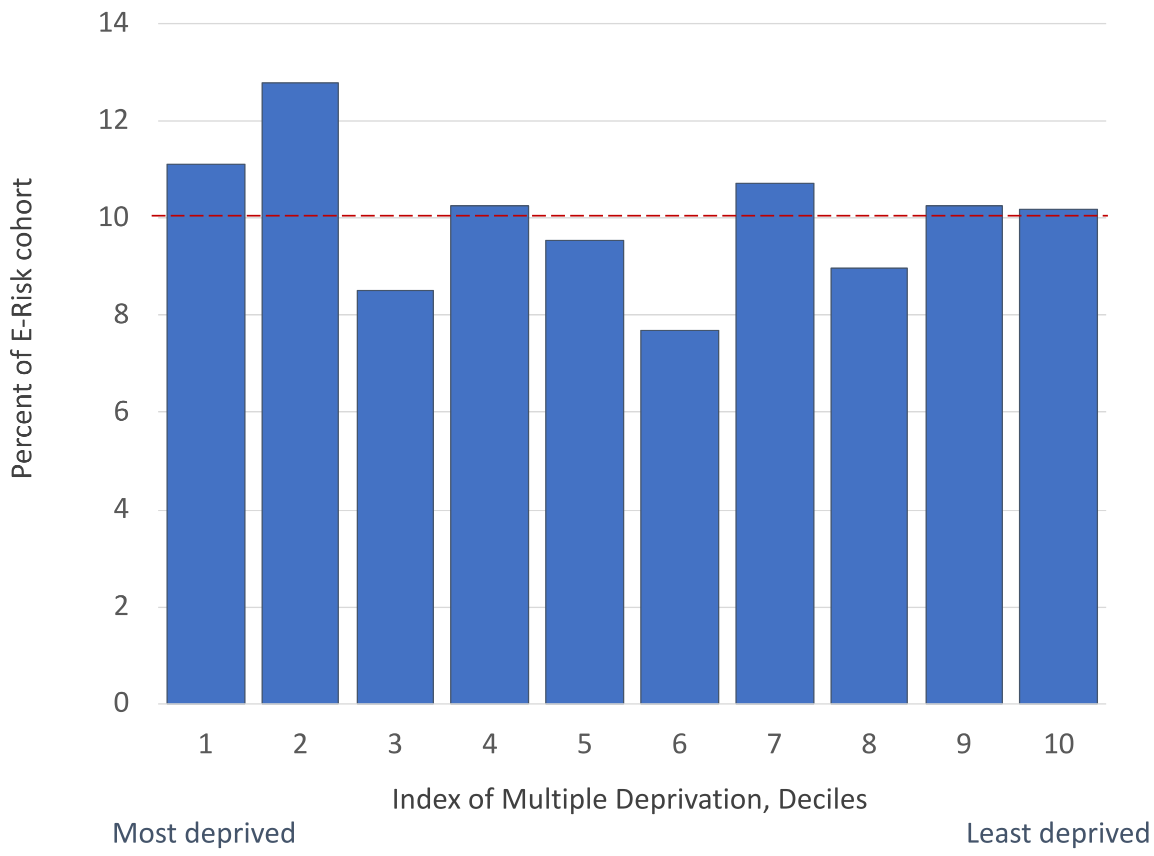

Note. The histogram shows E-Risk families' addresses are a near-perfect match to the deciles of the UK's 2015 Lower-layer Super Output Area (LSOA) Index of Multiple Deprivation (IMD) which averages 1,500 residents; approximately 10% of the cohort fills each of IMD's 10% bands for the UK.

© 2021 Baldwin JR et al. *JAMA Pediatrics*.

eFigure 2. Prevalence of ACEs in the E-Risk and Dunedin Cohorts in Comparison to the CDC ACEs Study

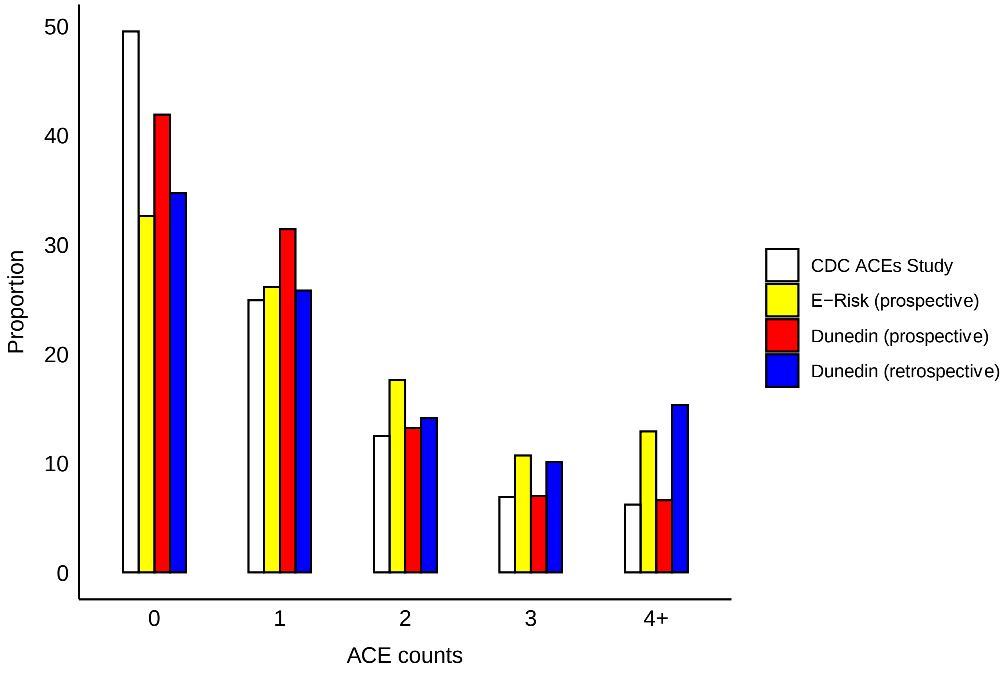

Note. The Figure shows the prevalence of ACEs in the E-Risk and Dunedin cohorts compared to the CDC ACEs Study[1]. Small differences reflect slightly lower levels of ACEs in the CDC ACEs Study relative to in the E-Risk and Dunedin cohorts, suggesting that under-detection of ACEs is not likely to be an issue in the E-Risk and Dunedin cohorts. Differences between the prevalences likely reflect differences in the assessment methods (e.g., repeated interviews and observations vs. a single questionnaire; prospective vs. retrospective assessment), informant (e.g. self-reports vs. parent reports/records), geographical locations (e.g., the USA, UK, or New Zealand) and ages.

© 2021 Baldwin JR et al. *JAMA Pediatrics*.

eFigure 3. Attrition Analysis in the Dunedin Study

A

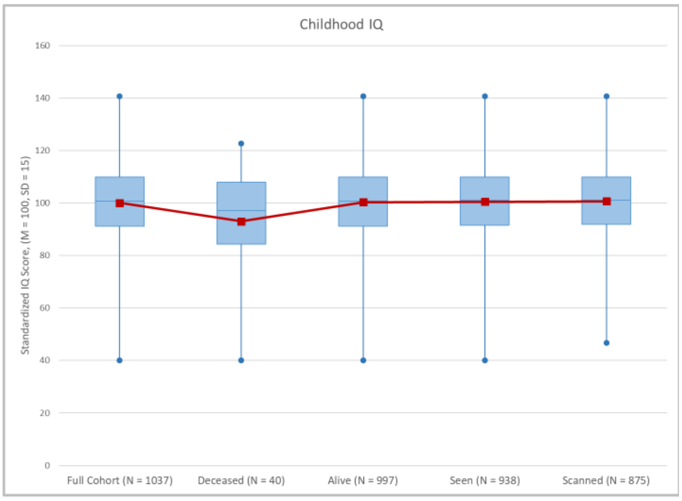

B

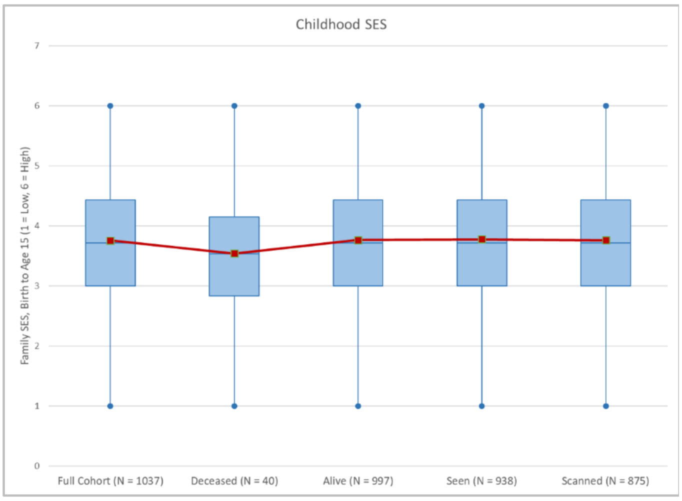

C

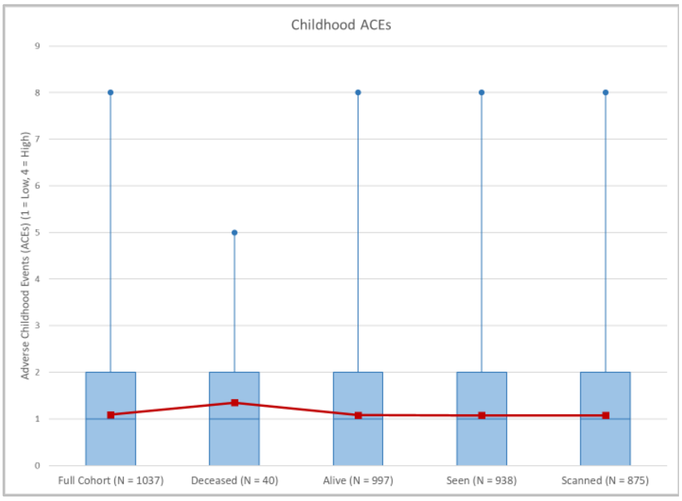

Abbreviations: IQ = intelligence quotient; SES = socioeconomic status; ACE = adverse childhood experiences. Note: We conducted an attrition analysis using childhood IQ (Panel A), childhood SES (Panel B), and prospectively measured ACEs (Panel C) to determine whether participants in the Phase 45 data collection were representative of the original cohort. No significant differences in childhood IQ were found between the full cohort, those still alive or those seen at Phase 45. Those who were deceased by the Phase 45 data collection had significantly lower childhood IQ's than those who were still alive ($t = 2.09$, $p = 0.04$). For childhood SES, no significant differences were found between the full cohort, those deceased, those alive, or those seen at Phase 45. For ACEs, no significant differences were found between the full cohort, those deceased, those alive, or those seen at Phase 4

© 2021 Baldwin JR et al. *JAMA Pediatrics*.

eFigure 4. Prevalence of Mental Health Problems in the E-Risk Cohort and in the Dunedin Cohort According to ACE Score

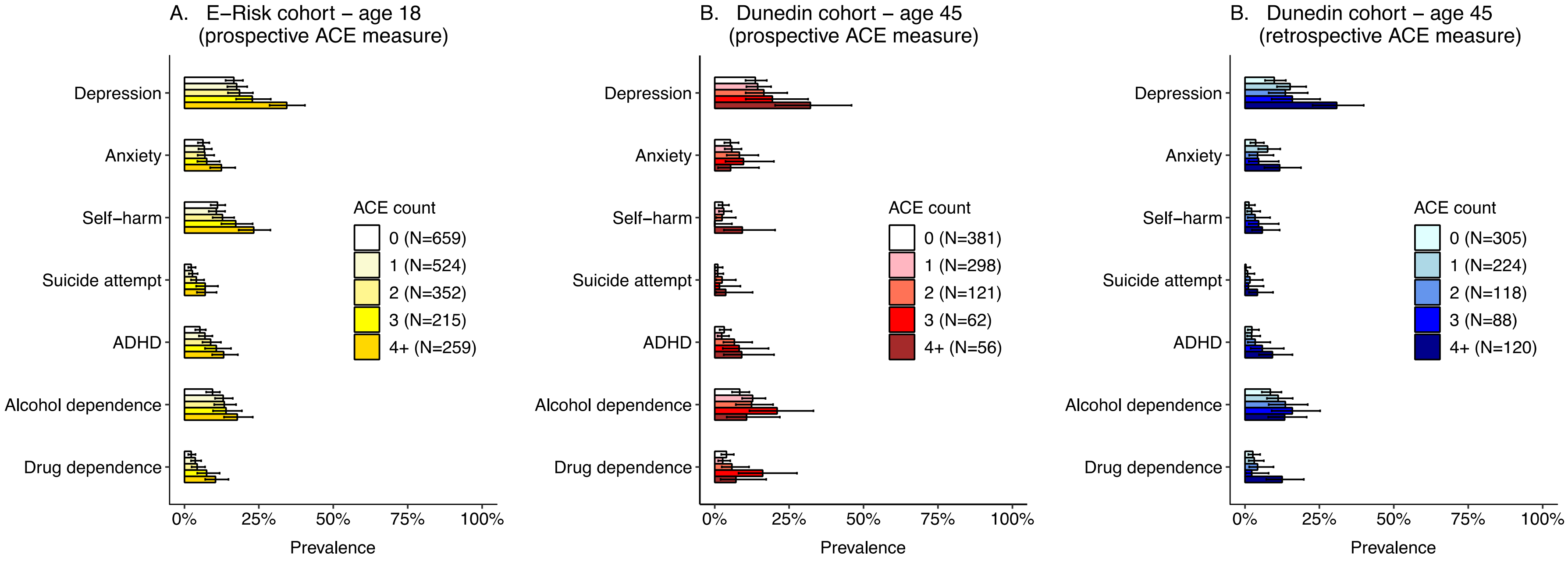

Abbreviations: ACE = adverse childhood experiences; ADHD = attention deficit hyperactivity disorder.
Note: The sample size as reported in the legend varies according to the health outcome (as reported fully in eTable 1).

© 2021 Baldwin JR et al. *JAMA Pediatrics*.

eFigure 5. Prevalence of Physical Health Problems in the E-Risk Cohort and in the Dunedin Cohort According to ACE Score

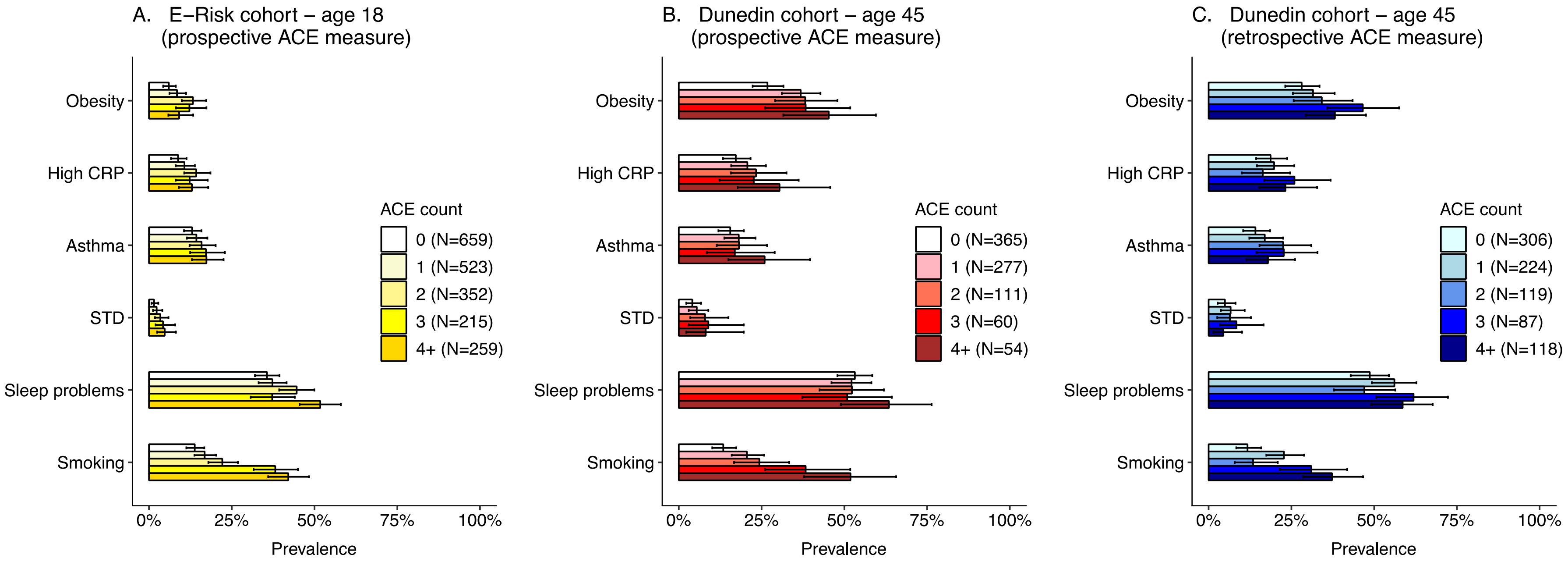

Abbreviations: ACE = adverse childhood experiences; CRP = C-reactive protein; STD = sexually transmitted disease.
Note: The sample size as reported in the legend varies according to the health outcome (as reported fully in eTable 1).

© 2021 Baldwin JR et al. *JAMA Pediatrics*.

eFigure 6. Predictive Accuracy for Individual Mental Health Problems Based on ACE Scores in the E-Risk and Dunedin Cohorts

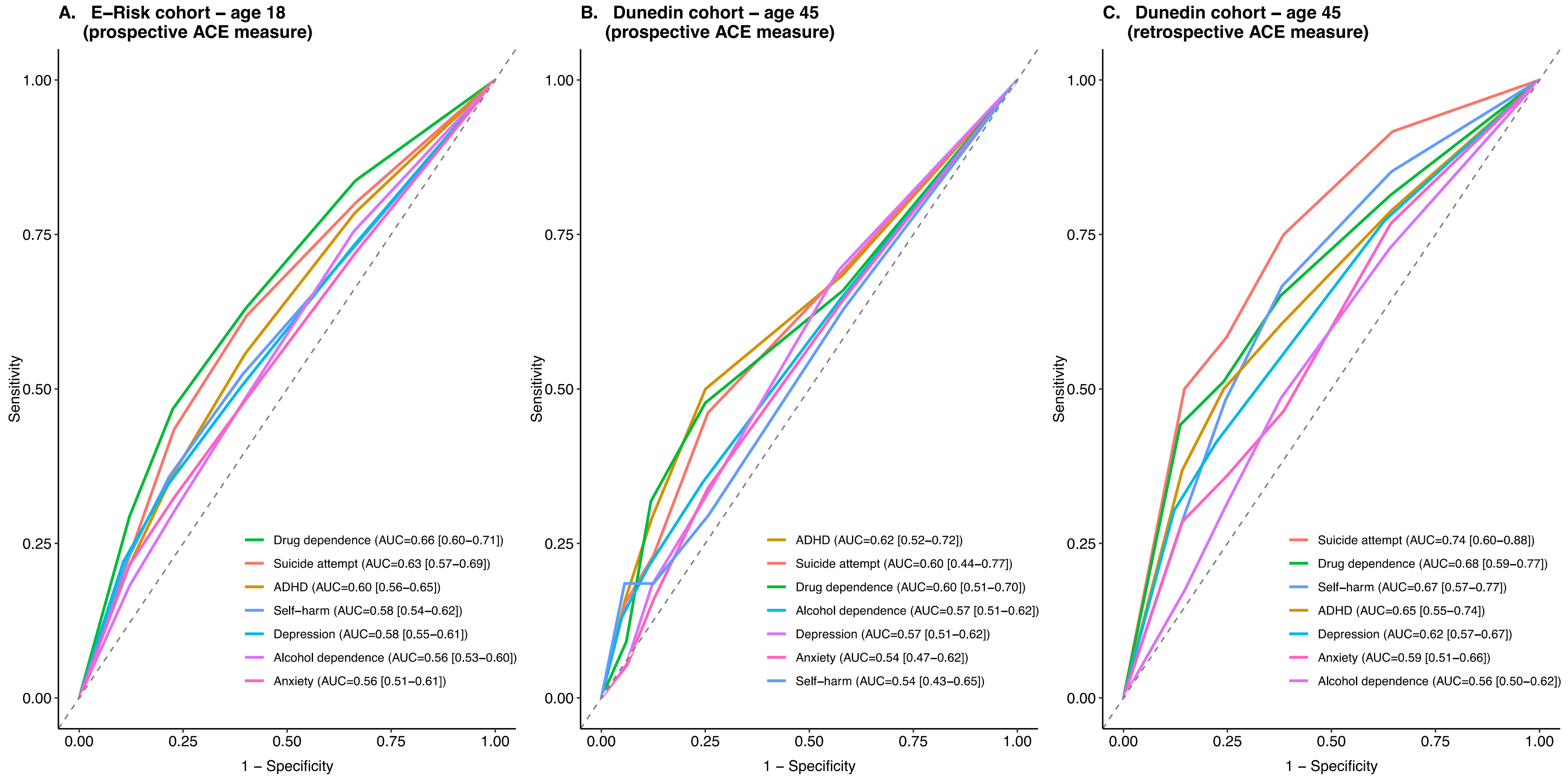

Abbreviations: ACE = adverse childhood experiences; ADHD = attention deficit hyperactivity disorder.
Note: The legend shows the area under the curve (AUC) value and 95% confidence intervals.

© 2021 Baldwin JR et al. *JAMA Pediatrics*.

eFigure 7. Predictive Accuracy for Individual Physical Health Problems Based on ACE Scores in the E-Risk and Dunedin Cohorts

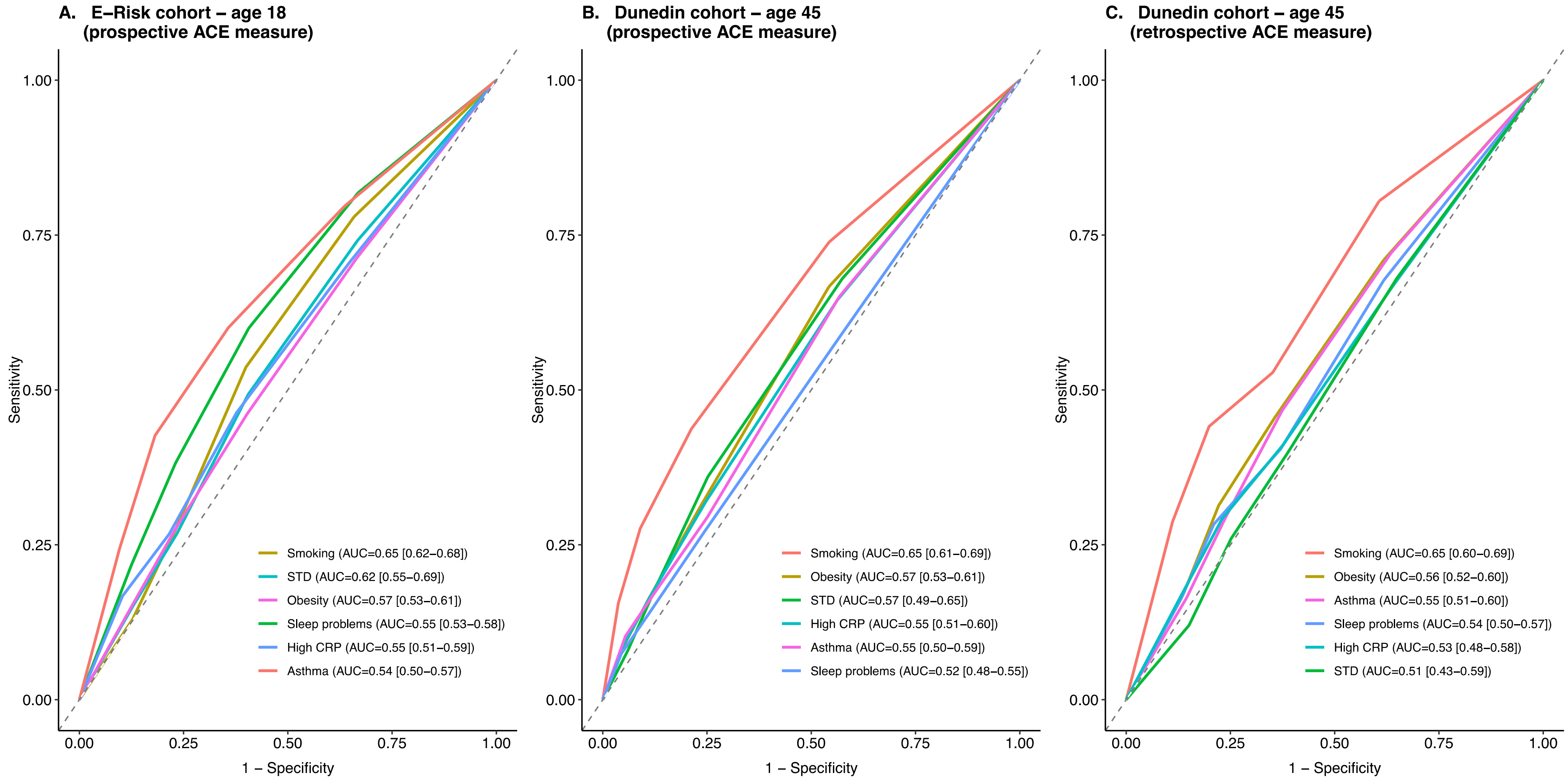

Abbreviations: ACE = adverse childhood experiences; CRP = C-reactive protein; STD = sexually transmitted disease.
Note: The legend shows the area under the curve (AUC) value and 95% confidence intervals.

© 2021 Baldwin JR et al. *JAMA Pediatrics*.

eReferences

1. Felitti VJ, Anda RF, Nordenberg D, et al. Relationship of childhood abuse and household dysfunction to many of the leading causes of death in adults: The Adverse Childhood Experiences (ACE) Study. *Am J Prev Med.* 1998;14(4):245-258.
2. Hughes K, Bellis MA, Hardcastle KA, et al. The effect of multiple adverse childhood experiences on health: a systematic review and meta-analysis. *Lancet Public Health.* 2017;2(8):e356-e366.
3. Trouton A, Spinath FM, Plomin R. Twins early development study (TEDS): a multivariate, longitudinal genetic investigation of language, cognition and behavior problems in childhood. *Twin Res.* 2002;5(5):444-448.
4. Moffitt TE, E-Risk Study Team. Teen-aged mothers in contemporary Britain. *J Child Psychol Psychiatry.* 2002;43(6):727-742.
5. Odgers CL, Caspi A, Russell MA, Sampson RJ, Arseneault L, Moffitt TE. Supportive parenting mediates neighborhood socioeconomic disparities in children's antisocial behavior from ages 5 to 12. *Dev Psychopathol.* 2012;24(3):705-721.
6. Dodge KA, Bates JE, Pettit GS. Mechanisms in the cycle of violence. *Science.* 1990;250(4988):1678-1683.
7. Lansford JE, Dodge KA, Pettit GS, Bates JE, Crozier J, Kaplow J. A 12-year prospective study of the long-term effects of early child physical maltreatment on psychological, behavioral, and academic problems in adolescence. *Arch Pediatr Adolesc Med.* 2002;156(8):824-830.
8. Straus MA. Measuring intrafamily conflict and violence: The Conflict Tactics (CT) scales. In: Straus MA, Gelles RG, eds. *Physical violence in American families: risk factors and adaptations to violence in 8,145 families* New Brunswick, NJ: Transaction Press; 1990:403-424.
9. Achenbach TM. *Manual for the young adult self-report and young adult behavior checklist.* University of Vermont, Department of Psychiatry; 1997.
10. Weissman MM, Wickramaratne P, Adams P, Wolk S, Verdeli H, Olfson M. Brief screening for family psychiatric history: the family history screen. *Arch Gen Psychiatry.* 2000;57(7):675-682.
11. Milne BJ, Caspi A, Harrington H, Poulton R, Rutter M, Moffitt TE. Predictive value of family history on severity of illness: the case for depression, anxiety, alcohol dependence, and drug dependence. *Arch Gen Psychiatry.* 2009;66(7):738-747.
12. Bradley RH, Caldwell BM. Home observation for measurement of the environment: a validation study of screening efficiency. *Am J Ment Defic.* 1977;81(5):417-420.
13. Danese A, Moffitt TE, Arseneault L, et al. The origins of cognitive deficits in victimized children: implications for neuroscientists and clinicians. *Am J Psychiatry.* 2017;174(4):349-361.
14. Beckley AL, Caspi A, Arseneault L, et al. The developmental nature of the victim-offender overlap. *Journal of Developmental and Life-course Criminology.* 2018;4(1):24-49.
15. Felitti VJ, Anda RF, Nordenberg D, et al. Relationship of childhood abuse and household dysfunction to many of the leading causes of death in adults. *Am J Prev Med.* 1998;14(4):245-258.
16. Purewal SK, Bucci M, Gutiérrez Wang L, et al. Screening for adverse childhood experiences (ACEs) in an integrated pediatric care model. *Zero to Three.* 2016;37(1):10-17.
17. Newbury JB, Arseneault L, Moffitt TE, et al. Measuring childhood maltreatment to predict early-adult psychopathology: comparison of

© 2021 Baldwin JR et al. *JAMA Pediatrics.*

prospective informant-reports and retrospective self-reports. *J Psychiatr Res.* 2018;96:57-64.
18. Baldwin JR, Arseneault A, Caspi A, et al. Adolescent victimization and self-injurious thoughts and behaviors: A genetically sensitive cohort study. *J Am Acad Child Adolesc Psychiatry.* 2019;58(5):506-513.
19. Robins L, Cottler L, Bucholz K, Compton W. *Diagnostic interview schedule for DSM-IV.* St Louis: Washington University; 1995.
20. Baldwin JR, Arseneault L, Caspi A, et al. Childhood victimization and inflammation in young adulthood: A genetically sensitive cohort study. *Brain, Behavior, and Immunity.* 2018;67:211-217.
21. Pearson TA, Mensah GA, Alexander RW, et al. Markers of inflammation and cardiovascular disease. *Circulation.* 2003;107(3):499-511.
22. Pearson TA, Mensah GA, Alexander RW, et al. Markers of inflammation and cardiovascular disease application to clinical and public health practice: a statement for healthcare professionals from the centers for disease control and prevention and the American Heart Association. *Circulation.* 2003;107(3):499-511.
23. Murphy TM, Wong CC, Arseneault L, et al. Methylomic markers of persistent childhood asthma: a longitudinal study of asthma-discordant monozygotic twins. *Clin Epigenetics.* 2015;7(1):130.
24. Ramrakha S, Bell ML, Paul C, Dickson N, Moffitt TE, Caspi A. Childhood behavior problems linked to sexual risk taking in young adulthood: A birth cohort study. *J Am Acad Child Adolesc Psychiatry.* 2007;46(10):1272-1279.
25. Johnson AM, Wadsworth J, Wellings K, Field J. *Sexual attitudes and lifestyles.* Blackwell Scientific; 1994.
26. Buysse DJ, Reynolds III CF, Monk TH, Berman SR, Kupfer DJ. The Pittsburgh Sleep Quality Index: a new instrument for psychiatric practice and research. *Psychiatry Res.* 1989;28(2):193-213.
27. Trzesniewski KH, Moffitt TE, Caspi A, Taylor A, Maughan B. Revisiting the association between reading achievement and antisocial behavior: New evidence of an environmental explanation from a twin study. *Child Development.* 2006;77(1):72-88.
28. Achenbach T. *Manual for the Child Behaviour Checklist and 1991 profile.* Burlington, VT: Department of Psychiatry, University of Vermont; 1991.
29. Achenbach T. *Manual for the Teacher's Report Form and 1991 Profile.* Burlington, VT: Department of Psychiatry, University of Vermont; 1991.
30. Caspi A, Moffitt TE, Thornton A, et al. The life history calendar: a research and clinical assessment method for collecting retrospective event-history data. *Int J Methods Psychiatr Res.* 1996.
31. Baldwin JR, Arseneault L, Odgers C, et al. Childhood bullying victimization and overweight in young adulthood: A cohort study. *Psychosom Med.* 2016;78(9):1094-1103.
32. Poulton R, Moffitt TE, Silva PA. The Dunedin Multidisciplinary Health and Development Study: overview of the first 40 years, with an eye to the future. *Soc Psychiatry Psychiatr Epidemiol.* 2015;50(5):679-693.
33. Jenny C. Medicine discovers child abuse. *JAMA.* 2008;300(23):2796-2797.
34. Bernstein D, Fink L. CTQ: Childhood Trauma Questionnaire: a retrospective self-report. *San Antonio, TX: Psychological Corp.* 1998.
35. Robins LN, Helzer J, Croughan J, Ratcliff KS. Diagnostic interview schedule. *Arch Gen Psychiatry.* 1981;38:381-389.
36. American Psychiatric Association. *Diagnostic and statistical manual of mental disorders (5th ed.).* Washington, DC: American Psychiatric Association; 2013.
37. Castillo R, Carlat D, Millon T, et al. *Diagnostic and statistical manual of mental disorders.* Washington, DC: American Psychiatric Association Press; 2007.

© 2021 Baldwin JR et al. *JAMA Pediatrics.*

38. Paul C, Van Roode T, Herbison P, Dickson N. Longitudinal study of self-reported sexually transmitted infection incidence by gender and age up to age thirty-two years. *Sexually Transmitted Diseases.* 2009;36(2):63-69.
39. McGee R, Williams SM, Silva PA. Factor structure and correlates of ratings of inattention, hyperactivity, and antisocial behavior in a large sample of 9-yr-old children from the general population. *J Consult Clin Psychol.* 1985;53(4):480.
40. Belsky DW, Caspi A, Israel S, Blumenthal JA, Poulton R, Moffitt TE. Cardiorespiratory fitness and cognitive function in midlife: neuroprotection or neuroselection? *Ann Neurol.* 2015;77(4):607-617.
41. Bayley N. *Manual for the Bayley scales of infant development.* Psychological Corporation; 1969.
42. MacCarthy D. *Manual for the McCarthy scales of children's abilities.* Psychological Corporation; 1972.
43. Arnheim D, Sinclair SW. *The Clumsy Child.* St Louis Mo: VC Mosby Co; 1974.
44. Belsky DW, Moffitt TE, Houts R, et al. Polygenic risk, rapid childhood growth, and the development of obesity: evidence from a 4-decade longitudinal study. *Arch Pediatr Adolesc Med.* 2012;166(6):515-521.
45. Williams S, Poulton R. Birth size, growth, and blood pressure between the ages of 7 and 26 years: failure to support the fetal origins hypothesis. *Am J Epidemiol.* 2002;155(9):849-852.
46. Sears MR, Greene JM, Willan AR, et al. A longitudinal, population-based, cohort study of childhood asthma followed to adulthood. *New England Journal of Medicine.* 2003;349(15):1414-1422.
47. Reuben A, Caspi A, Belsky DW, et al. Association of childhood blood lead levels with cognitive function and socioeconomic status at age 38 years and with IQ change and socioeconomic mobility between childhood and adulthood. *JAMA.* 2017;317(12):1244-1251.
48. Idler EL, Benyamini Y. Self-rated health and mortality: a review of twenty-seven community studies. *J Health Soc Behav.* 1997:21-37.

© 2021 Baldwin JR et al. *JAMA Pediatrics.*
